# Supplementary material for: Evolution of foraging behaviour induces variable complexity-stability relationships in mutualist-exploiter-predator communities
Source: PLoS Comput Biol. 2025 Jul 9;21(7):e1013245. doi: 10.1371/journal.pcbi.1013245 (PMC12240360; doi:10.1371/journal.pcbi.1013245)
Supplement: S1 Appendix — (DOCX) [file pcbi.1013245.s001.docx]

**S1 Appendix---Population chaos in the four-species MEST community**

In the four-species MEST community, our adaptive network model exhibits a complex dynamic behaviour as the intensity (*g*) of foraging adaptation increases (Fig A-C).


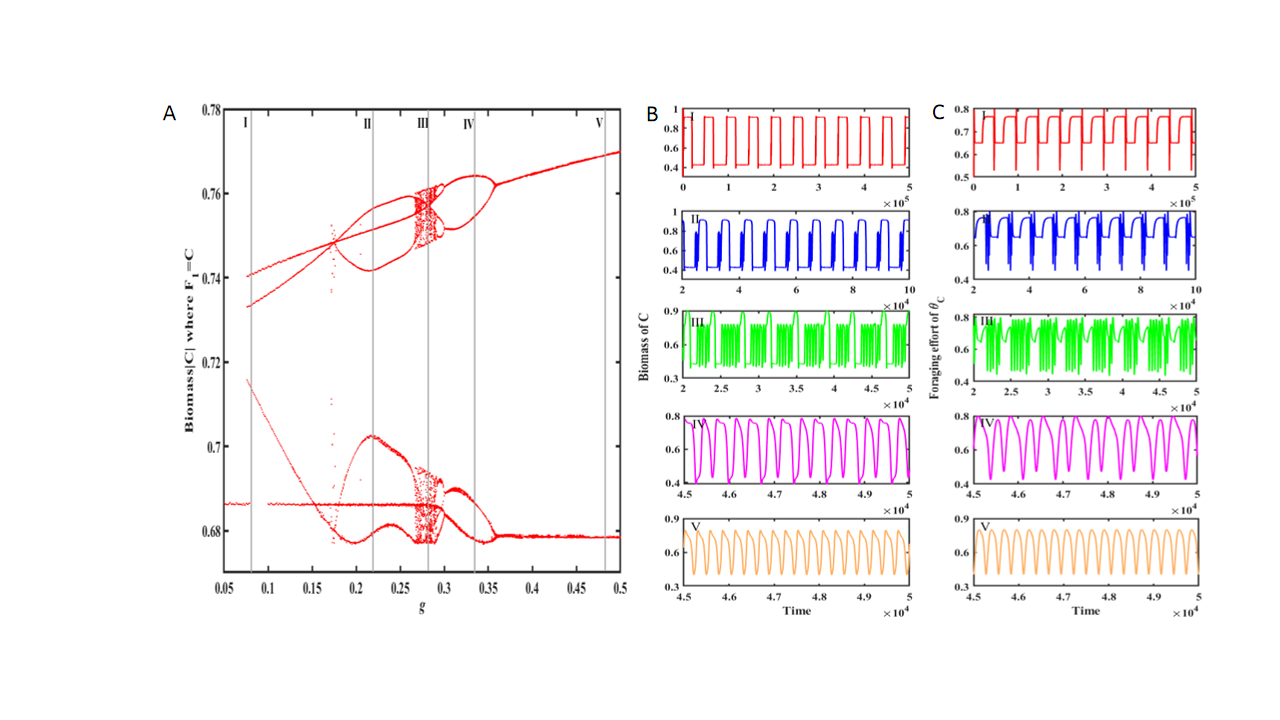


Fig A. Population dynamics of the specialist predator (*C*) and preference trait dynamics ($\theta_{C}$) vary with the adaptation intensity (*g*; case I、II、IV、V: regular periodic oscillation; case III: chaos). (A) bifurcation diagram; (B) population biomss of the specialist predator changes with time; (C) foraging effort $\theta_{C}$ changes with time.

As *g* increases, both population dynamics of the specialist predator (*C*) and preference trait dynamics ($\theta_{C}$) evolve from simple periodic behaviour (*g*=0.08, case I; Fig A), period-doubling oscillations (*g*=0.22, case II; Fig A) into chaos (*g*=0.28, case III; Fig A). As the value of *g* is increased, both population dynamics of *F_1_* and the trait dynamics will change from chaos to period-doubling oscillations (*g*=0.34, case IV; Fig A) and simple cycles (*g*=0.48, case V; Fig A). Similarly, as *g* increases, population dynamics of the top predator (*P*) evolves from simple periodic behaviour (*g*=0.08, case I; Fig B), period-doubling oscillations (*g*=0.22, case II; Fig B) into chaos (*g*=0.28, case III; Fig B). As the value of *g* is increased, population dynamics of *P* will change from chaos to period-doubling oscillations (*g*=0.34, case IV; Fig B) and simple cycles (*g*=0.48, case V; Fig B); moreover, for the mutualist (*F_0_*), these similar patterns (e.g., regular periodic oscillation and chaos) can also be obtained (Fig C).


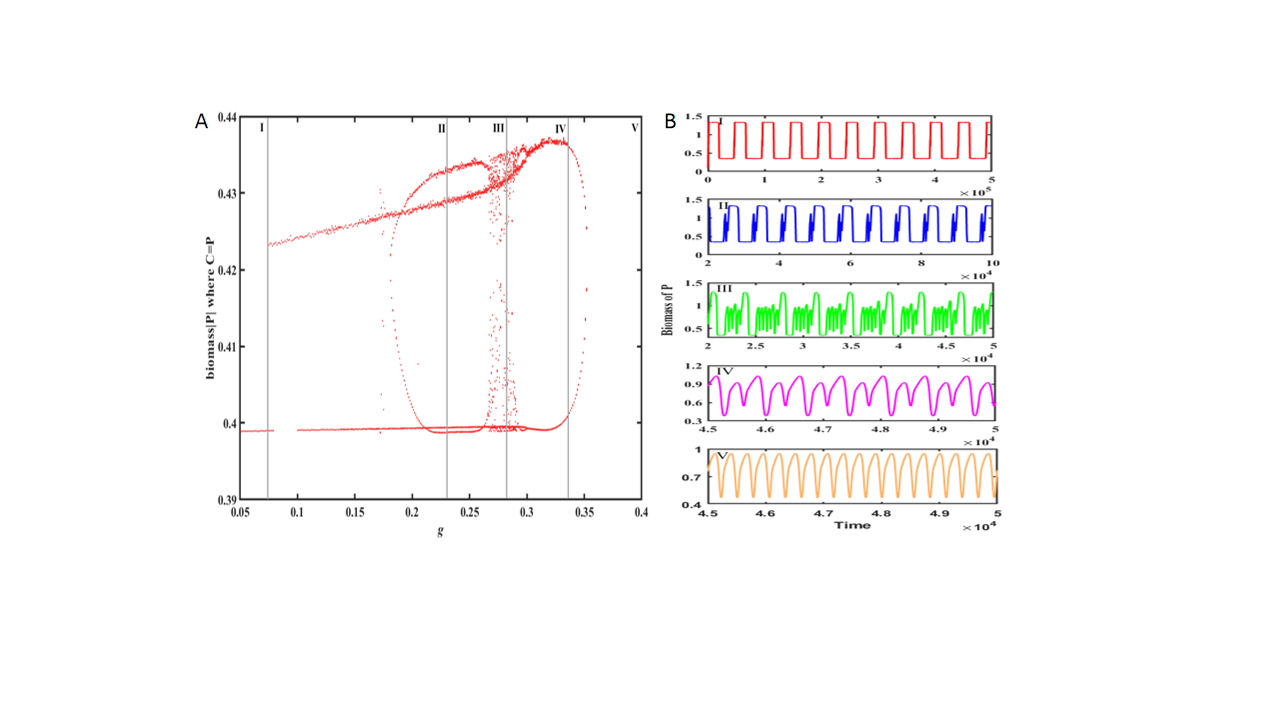


Fig B. Population dynamics of the top predator (*P*) changes with the adaptation intensity (*g*; case I、II、IV、V: regular periodic oscillation; case III: chaos). (A) bifurcation diagram; (B) population biomss of the top predator changes with time.


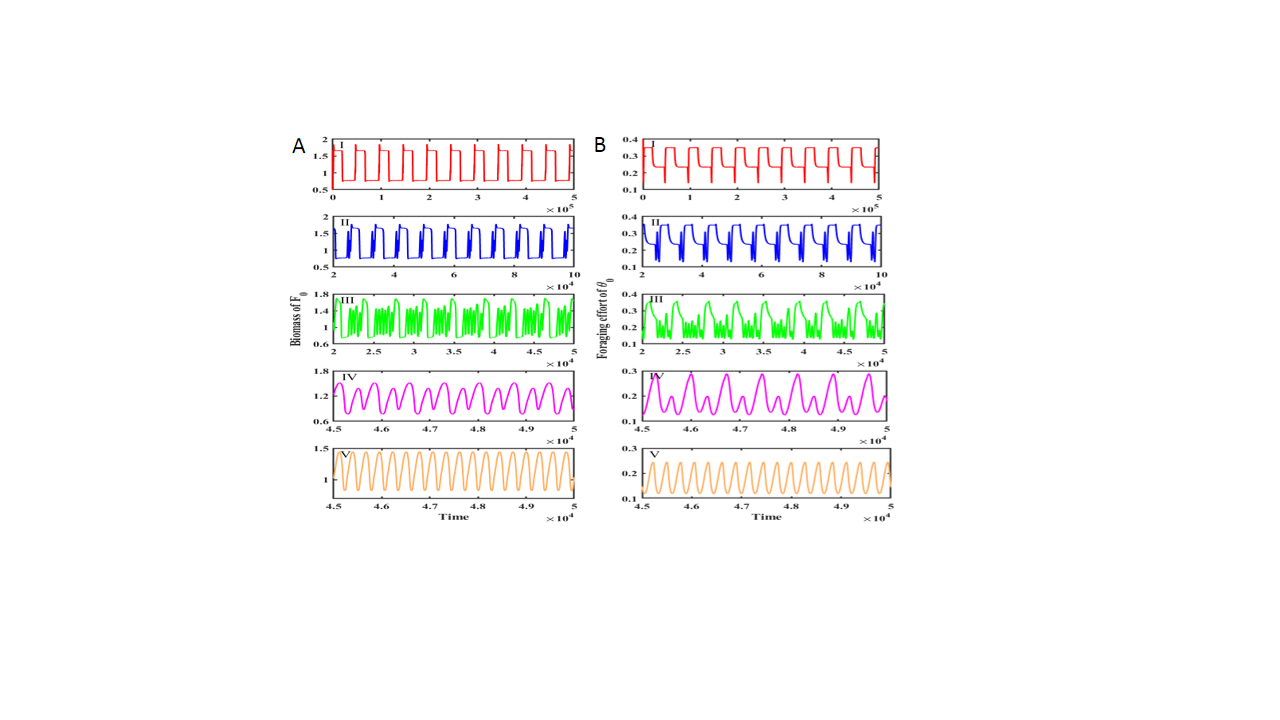


Fig C. Population dynamics of the mutualist (F0) and preference trait (*θ_0_*) dynamics vary with the adaptation intensity (g; case I、II、IV、V: regular periodic oscillation; case III: chaos). (A) population biomss of the mutualist changes with time; (B) foraging effort *θ_0_* changes with time.
