# Supplementary material for: Evolution of foraging behaviour induces variable complexity-stability relationships in mutualist-exploiter-predator communities
Source: PLoS Comput Biol. 2025 Jul 9;21(7):e1013245. doi: 10.1371/journal.pcbi.1013245 (PMC12240360; doi:10.1371/journal.pcbi.1013245)
Supplement: S2 Appendix — (DOCX) [file pcbi.1013245.s002.docx]

**S2 Appendix---Sensitivity analyses of model parameters in the four-species MEST community**

In the four-species MEST community, to analyze the influence of model parameters on state variables and understand parameter sensitivity across continuous time intervals, we employed the Latin Hypercube Sampling-Partial Rank Correlation Coefficient (LHS-PRCC) method for sensitivity analyses (Marino et al., 2008). This methodology provides a quantitative framework for identifying critical system parameters and their dynamic roles in governing system behaviour. During parameter sampling, a uniform distribution was selected as the prior distribution with a sampling size of 1,000 iterations. Model parameters were designated as input variables, with their ranges set to ±15%–20% of baseline values, while state variables were treated as output variables. A positive PRCC value indicates that the parameter exerts a promoting effect on the state variable, whereas a negative value suggests an inhibitory effect (Table A and Fig A-B).

Table A. The PRCC value of model parameters corresponding to state variables at t=2000

| **Parameters** | $\boldsymbol{F}_{\boldsymbol{0}}$ | | $\boldsymbol{F}_{\boldsymbol{1}}$ | | $\boldsymbol{C}$ | | $\boldsymbol{P}$ | | $\boldsymbol{\theta}_{\boldsymbol{0}}$ | | $\boldsymbol{\theta}_{\boldsymbol{1}}$ | |
| --- | --- | --- | --- | --- | --- | --- | --- | --- | --- | --- | --- | --- |
|  | **PRCCs** | **p-value** | **PRCCs** | **p-value** | **PRCCs** | **p-value** | **PRCCs** | **p-value** | **PRCCs** | **p-value** | **PRCCs** | **p-value** |
| $\boldsymbol{r}_{\boldsymbol{0}}$ | **0.7488** | **P<0.01** | **-0.15736** | **P<0.01** | **0.67564** | **P<0.01** | **0.70268** | **P<0.01** | **0.63828** | **P<0.01** | **-0.768** | **P<0.01** |
| $\boldsymbol{r}_{\boldsymbol{1}}$ | **-0.50852** | **P<0.01** | **0.59052** | **P<0.01** | **-0.41164** | **P<0.01** | **-0.35919** | **P<0.01** | **-0.59564** | **P<0.01** | **0.76348** | **P<0.01** |
| $\boldsymbol{\beta}$ | **0.44136** | **P<0.01** | **-0.67244** | **P<0.01** | **0.46975** | **P<0.01** | **0.44677** | **P<0.01** | **0.34446** | **P<0.01** | **-0.54808** | **P<0.01** |
| $\boldsymbol{u}_{\boldsymbol{0}}$ | **-0.42011** | **P<0.01** | **0.33251** | **P<0.01** | **-0.21443** | **P<0.01** | **-0.18316** | **P<0.01** | **0.64944** | **P<0.01** | **0.27867** | **P<0.01** |
| $\boldsymbol{u}_{\boldsymbol{1}}$ | **0.15778** | **P<0.01** | **-0.44621** | **P<0.01** | **0.13584** | **P<0.01** | **0.16022** | **P<0.01** | **0.23031** | **P<0.01** | **-0.29209** | **P<0.01** |
| $\boldsymbol{u}_{\boldsymbol{C}}$ | **0.37005** | **P<0.01** | **-0.18589** | **P<0.01** | **-0.10529** | **P<0.01** | **0.37654** | **P<0.01** | **0.13117** | **P<0.01** | **-0.35845** | **P<0.01** |
| $\boldsymbol{e}_{\boldsymbol{C}}$ | -0.03437 | 0.28200 | **0.09989** | **P<0.01** | 0.08007 | 0.01208 | **0.08702** | **P<0.01** | **-0.51432** | **P<0.01** | 0.00273 | 0.93185 |
| $\boldsymbol{e}_{\boldsymbol{P}}$ | 0.05763 | 0.07105 | **-0.12041** | **P<0.01** | -0.07715 | 0.0156 | **0.21684** | **P<0.01** | **0.39218** | **P<0.01** | **-0.12421** | **P<0.01** |
| $\boldsymbol{d}_{\boldsymbol{0}}$ | **-0.12282** | **P<0.01** | -0.04216 | 0.18685 | **-0.13142** | **P<0.01** | **-0.14635** | **P<0.01** | -0.0582 | 0.06829 | **0.14434** | **P<0.01** |
| $\boldsymbol{d}_{\boldsymbol{1}}$ | 0.07601 | 0.01721 | **-0.08698** | **P<0.01** | 0.01354 | 0.67168 | 0.04664 | 0.14417 | **0.13457** | **P<0.01** | **-0.15553** | **P<0.01** |
| $\boldsymbol{d}_{\boldsymbol{C}}$ | -0.02229 | 0.48536 | -0.06090 | 0.05643 | -0.03916 | 0.22015 | -0.01516 | 0.63512 | 0.06989 | 0.02852 | 0.0151 | 0.63664 |
| $\boldsymbol{d}_{\boldsymbol{P}}$ | 0.00945 | 0.76736 | 0.02796 | 0.38143 | -0.03002 | 0.3473 | **-0.20979** | **P<0.01** | **-0.21808** | **P<0.01** | **0.10538** | **P<0.01** |
| $\boldsymbol{\alpha}_{\boldsymbol{0}}$ | **-0.39972** | **P<0.01** | **0.18549** | **P<0.01** | **-0.35562** | **P<0.01** | **-0.36258** | **P<0.01** | **-0.3063** | **P<0.01** | **0.39584** | **P<0.01** |
| $\boldsymbol{\alpha}_{\boldsymbol{C}}$ | 0.05713 | 0.07355 | -0.07907 | 0.01319 | -0.01586 | 0.61963 | -0.02634 | 0.40969 | **0.3745** | **P<0.01** | **-0.0121** | **P<0.01** |
| $\boldsymbol{\alpha}_{\boldsymbol{P}}$ | **-0.08685** | **P<0.01** | **0.17048** | **P<0.01** | 0.01867 | 0.55903 | **-0.28423** | **P<0.01** | **-0.40168** | **P<0.01** | **0.14396** | **P<0.01** |
| $\boldsymbol{q}$ | -0.01451 | 0.64974 | 0.05618 | 0.07845 | -0.01074 | 0.73674 | -0.03195 | 0.31727 | **0.11694** | **P<0.01** | **-0.14253** | **P<0.01** |
| $\boldsymbol{a}_{\boldsymbol{C}}$ | **-0.46027** | **P<0.01** | **0.26262** | **P<0.01** | **-0.219** | **P<0.01** | **-0.23335** | **P<0.01** | **-0.81322** | **P<0.01** | 0.3212 | 0.7048 |
| $\boldsymbol{g}$ | 0.02727 | 0.39327 | 0.02979 | 0.35106 | -0.0628 | 0.04913 | -0.01456 | 0.64865 | -0.04746 | 0.13727 | -0.02583 | 0.41872 |

In table A, the magnitude of the PRCC absolute value reflects the parameter’s influence strength: (i) weak and statistically insignificant association between input parameter and output variable (i.e., |PRCC|∈[0, 0.2]); (ii) moderate correlation between the variables (i.e., |PRCC|∈(0.2, 0.4]) and (iii) strong correlation between input parameter and output variable (i.e., |PRCC|∈(0.4, 1]). Furthermore, statistical significance was evaluated through p-values, that is, when *p* > 0.01, the parameter’s impact on the state variable is not statistically significant; while the parameter exhibits essentially no measurable influence on the state variable if *p* > 0.05.

As shown in Fig A, with the increase of simulation time (i.e., t > 400), the influence of model parameters on state variables will tend to stabilize to constant values. Therefore, by setting t = 2000, we can obtain the significant influence of key parameters on the state variables (Fig B). In Fig B(A), parameters $r_{0}$, $\beta$, $u_{C}$, $\alpha_{0}$, $u_{0}$, $a_{C}$ and $r_{1}$ exhibit pronounced influences on F0 (*p* < 0.01; table A). Specifically, parameters $r_{0}$, $\beta$ and $u_{C}$ promote the increase of F0, with their effect magnitudes ranked as $\left| PRCC\left( r_{0} \right) \right|>\left| PRCC\left( \beta\right) \right|>\left| PRCC\left( u_{C} \right) \right|$; while the parameters $\alpha_{0}$, $u_{0}$, $a_{C}$ and $r_{1}$ inhibit the growth of F0, following the hierarchy $\left| PRCC\left( r_{1} \right) \right|>\left| PRCC\left( a_{C} \right) \right|>\left| PRCC\left( u_{0} \right) \right|>\left| PRCC\left( \alpha_{0} \right) \right|$. In Fig B(B), the parameters $u_{0}$, $a_{C}$ and $r_{1}$ promote the increase of F1, with the order of effect magnitude being $\left| PRCC\left( r_{1} \right) \right|>\left| PRCC\left( u_{0} \right) \right|>\left| PRCC\left( a_{C} \right) \right|$; while the parameters $u_{1}$ and β inhibit the increase of F1, where $\left| PRCC\left( \beta\right) \right|>\left| PRCC\left( u_{1} \right) \right|$. In Fig B(C), the parameters $r_{0}$​, β, and $u_{1}$ promote the increase of C, with the magnitude of effects following $\left| PRCC\left( r_{0} \right) \right|>\left| PRCC\left( \beta\right) \right|>\left| PRCC\left( u_{1} \right) \right|$; while the parameters $\alpha_{0}$ and $r_{1}$ inhibit the increase of C, where $\left| PRCC\left( r_{1} \right) \right|>\left| PRCC\left( \alpha_{0} \right) \right|$. In Fig B(D), the parameters $r_{0}$, $\beta$, $u_{C}$, $e_{P}$, and $u_{1}$ promote the increase of P, with the magnitude of effects following $\left| PRCC\left( r_{0} \right) \right|>\left| PRCC\left( \beta\right) \right|>\left| PRCC\left( u_{C} \right) \right|>\left| PRCC\left( e_{P} \right) \right|>\left| PRCC\left( u_{1} \right) \right|$; while the parameters $d_{P}$, $a_{C}$, $\alpha_{P}$, $r_{1}$, and $\alpha_{0}$ inhibit the increase of P, where $\left| PRCC\left( \alpha_{0} \right) \right|>\left| PRCC\left( r_{1} \right) \right|>\left| PRCC\left( \alpha_{P} \right) \right|>\left| PRCC\left( a_{C} \right) \right|>\left| PRCC\left( d_{P} \right) \right|$. In Fig B(E), the parameters $u_{0}$, $r_{0}$, $e_{P}$, $\alpha_{C}$, $\beta$, and $u_{1}$ promote the increase of $\theta_{0}$, with the magnitude of effects following$\left| PRCC\left( u_{0} \right) \right|>\left| PRCC\left( r_{0} \right) \right|>\left| PRCC\left( e_{P} \right) \right|>\left| PRCC\left( \alpha_{C} \right) \right|>\left| PRCC\left( \beta\right) \right|>\left| PRCC\left( u_{1} \right) \right|$; while the parameters $d_{P}$, $\alpha_{0}$, $\alpha_{P}$, $e_{C}$, $r_{1}$, and $a_{C}$ inhibit the increase of $\theta_{0}$, where $\left| PRCC\left( a_{C} \right) \right|>\left| PRCC\left( r_{1} \right) \right|>\left| PRCC\left( e_{C} \right) \right|>\left| PRCC\left( \alpha_{P} \right) \right|>\left| PRCC\left( \alpha_{0} \right) \right|>\left| PRCC\left( d_{P} \right) \right|$. In Fig B(F), the parameters $u_{1}$, $\alpha_{0}$, $r_{1}$, and $a_{C}$ promote the increase of $\theta_{1}$, with the magnitude of effects following $\left| PRCC\left( r_{1} \right) \right|>\left| PRCC\left( \alpha_{0} \right) \right|>\left| PRCC\left( a_{C} \right) \right|>\left| PRCC\left( u_{1} \right) \right|$; while the parameters $u_{C}$, $r_{0}$, and $\beta$ inhibit the increase of $\theta_{1}$, where $\left| PRCC\left( r_{0} \right) \right|>\left| PRCC\left( \beta\right) \right|>\left| PRCC\left( u_{c} \right) \right|$.


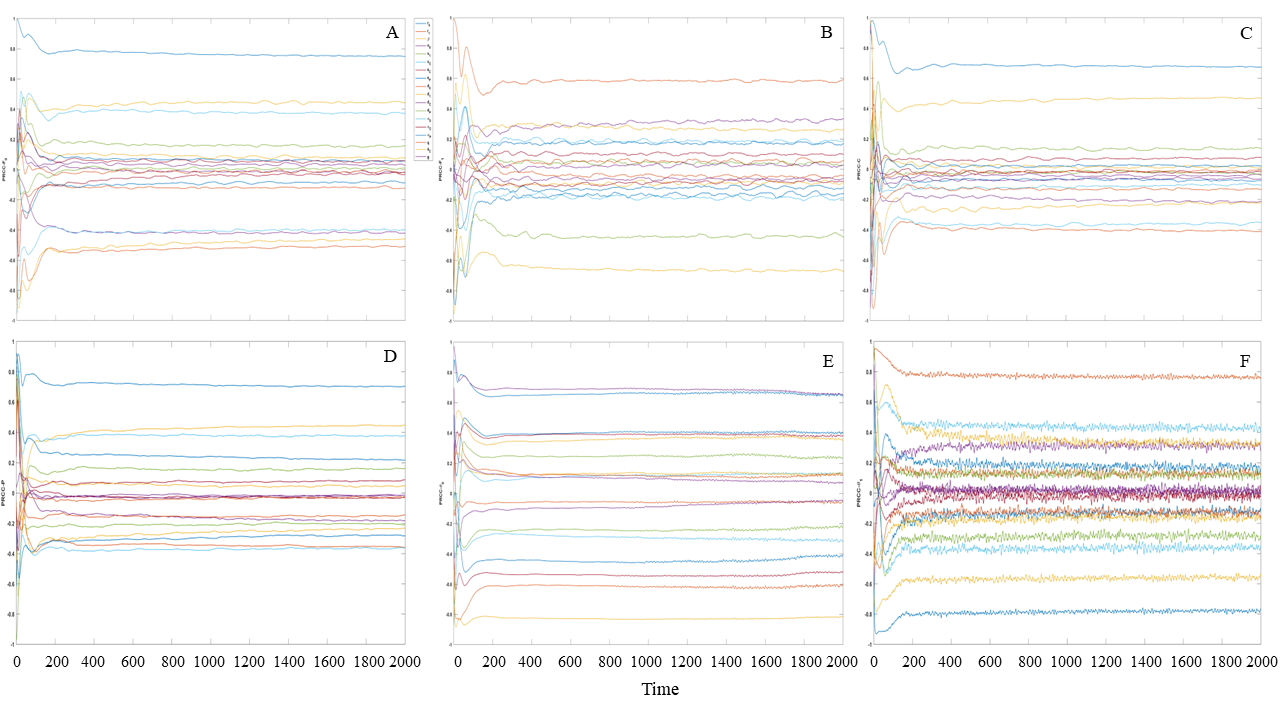


Fig A. Sensitivity of parameters to each state variable over continuous time interval. (A) PRCC-F0; (B) PRCC-F1; (C) PRCC-C; (D) PRCC-P; (E) PRCC-θ_0_; (F) PRCC-θ_1_.


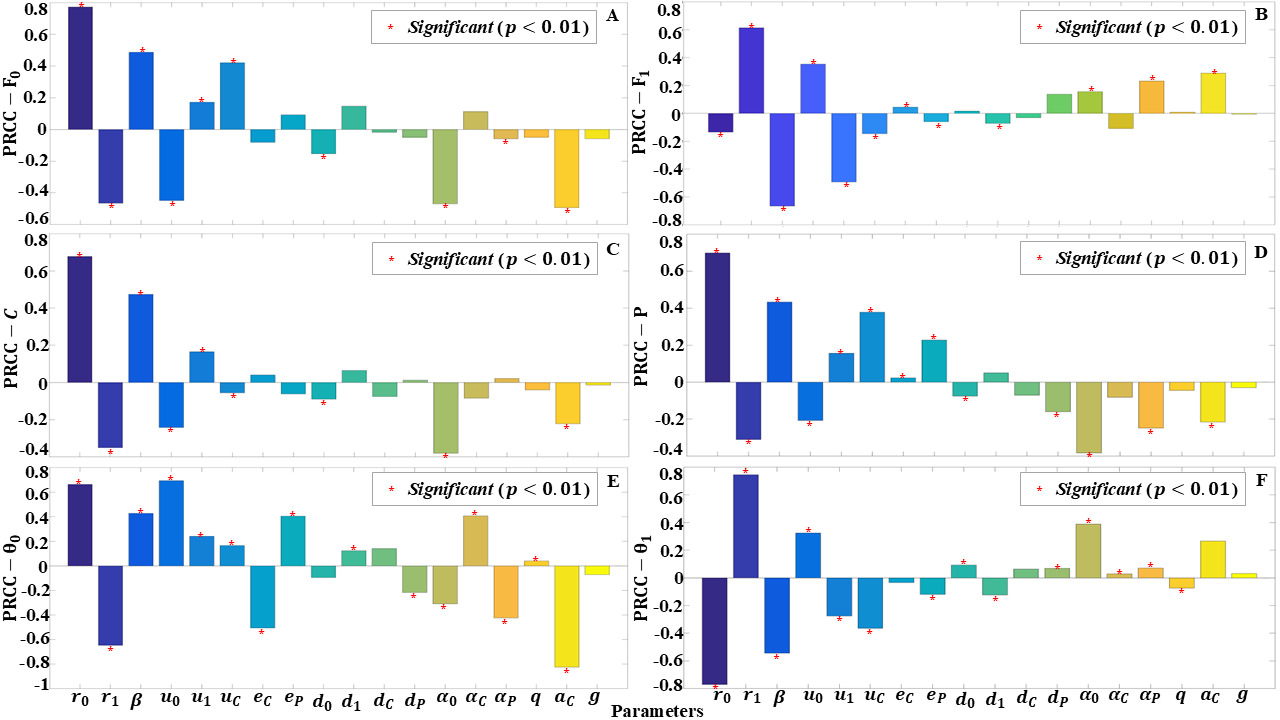


Fig B. Sensitivity analysis using PRCC at t=2000. (A) PRCC-F0; (B) PRCC-F1; (C) PRCC-C; (D) PRCC-P; (E) PRCC-θ_0_; (F) PRCC-θ_1_.

**References**

Marino, S., Hogue, I.B., Ray, C.J., Kirschner, D.E. A methodology for performing global uncertainty and sensitivity analysis in systems biology. Journal of Theoretical Biology. 2008;254(1):178-196.
