# Supplementary material for: Evolution of foraging behaviour induces variable complexity-stability relationships in mutualist-exploiter-predator communities
Source: PLoS Comput Biol. 2025 Jul 9;21(7):e1013245. doi: 10.1371/journal.pcbi.1013245 (PMC12240360; doi:10.1371/journal.pcbi.1013245)
Supplement: S3 Appendix — (DOCX) [file pcbi.1013245.s003.docx]

**S3 Appendix---Structural sensitivity analyses of** **mutualist-exploiter-predator communities with the basal resource**

Here, to analyze structural sensitivity of the mutualist-exploiter-predator (MEST) communities, we added the basal resource (R) into the four-species MEST community and established one new adaptive network model modified with carrying capacity as a function of density of interaction partners (May 1981; Addicott 1981; Wolin & Lawlor 1984):

$\frac{dR}{dt}=\underset{\begin{aligned} mutualism- \\ dependent \\ growth \end{aligned}}{\underbrace{r_{1}R\left( 1-\frac{R}{{F_{0}}/k} \right)}}-\underset{\begin{aligned} seed consumption \\ by F_{0} \end{aligned}}{\underbrace{mRF_{0}}}-\underset{\begin{aligned} seed consumption \\ by F_{1} \end{aligned}}{\underbrace{vRF_{1}}}$,

$\frac{dF_{0}}{dt}=mRF_{0}-\underset{\begin{aligned} interspeciﬁc \\ competition \end{aligned}}{\underbrace{\beta F_{0}F_{1}}}-\underset{\begin{aligned} foraged \\ by C \end{aligned}}{\underbrace{a_{C}CF_{0}}}-\underset{\begin{aligned} foraged \\ by P \end{aligned}}{\underbrace{\theta_{0}u_{0}PF_{0}}}-\underset{death}{\underbrace{d_{0}F_{0}}}-\underset{\begin{aligned} density- \\ dependent \\ death \end{aligned}}{\underbrace{\alpha_{0}F_{0}^{2}}}$,

$\frac{dF_{1}}{dt}=vRF_{1}-\beta F_{0}F_{1}-\underset{\begin{aligned} foraged \\ by P \end{aligned}}{\underbrace{\theta_{1}u_{1}F_{1}P}}-\underset{death}{\underbrace{d_{1}F_{1}}}-\underset{\begin{aligned} density- \\ dependent \\ death \end{aligned}}{\underbrace{\alpha_{1}F_{1}^{2}}}$,

$\frac{dC}{dt}=C\left[ \underset{\begin{aligned} growth caused \\ by consumption \\ of C \end{aligned}}{\underbrace{e_{C}a_{C}F_{0}}}-\underset{\begin{aligned} foraged \\ by P \end{aligned}}{\underbrace{\theta_{c}u_{C}P}}-\underset{\begin{aligned} mortality \\ rate \end{aligned}}{\underbrace{d_{C}}}-\underset{\begin{aligned} density- \\ dependent \\ death \end{aligned}}{\underbrace{\alpha_{C}C}} \right]$,

$\frac{dP}{dt}=P\left[ \underset{\begin{aligned} growth caused \\ by consumption \\ of all prey \end{aligned}}{\underbrace{e_{P}\left( \theta_{0}u_{0}F_{0}+\theta_{1}u_{1}F_{1}+\theta_{c}u_{c}C \right)}}-\underset{\begin{aligned} mortality \\ rate \end{aligned}}{\underbrace{d_{P}}}-\underset{\begin{aligned} density- \\ dependent \\ death \end{aligned}}{\underbrace{\alpha_{P}P}} \right]\equiv PW_{P}$,

$\frac{d\theta_{0}}{dt}=g\theta_{0}\left[ e_{P}u_{0}F_{0}-e_{P}\left( \theta_{0}u_{0}F_{0}+\theta_{1}u_{1}F_{1}+\theta_{c}u_{c}C \right) \right]$,

$\frac{d\theta_{1}}{dt}=g\theta_{1}\left[ e_{P}u_{1}F_{1}-e_{P}\left( \theta_{0}u_{0}F_{0}+\theta_{1}u_{1}F_{1}+\theta_{c}u_{c}C \right) \right]$,

$\frac{d\theta_{C}}{dt}=-\frac{d\theta_{0}}{dt}-\frac{d\theta_{1}}{dt}$.

When fixed the intensity (*g*=0.1) of foraging adaptations, while varying both the consumption rate (*u_1_*) and interspecific competition (*β*), we can obtain four food-web structures and their local stability (Fig A). First, stable coexistence (i.e., *Re*(*λ_max_*)<0) will not exist (*Re*(*λ_max_*)>0; Fig A(B)) when the top predator *P* does not prey on the mutualist *F0* ($\theta_{0}=0$); when *P* does not prey on *C* ($\theta_{C}=0$) but consume *F0* ($\theta_{0}\neq0$), stable coexistence presents at low consumption rate *u_1_* and interspecific competition *β* (*Re*(*λ_max_*)<0; Fig A(A)). Predation of *F0* by *P* promotes stable coexistence of species also presented in other food web structures (Fig A(D) and Fig A(A)). Specifically, when *P* does not prey on the mutualist *F_0_* and *C* ($\theta_{C}=0, \theta_{0}=0$), no stable coexistence exists in the system (*Re*(*λ_max_*)>0; Fig A(C)). Second, by comparing Fig A(B) and Fig A(D), we can intuitively find that compared to *P* does not prey on *F_0_* ($\theta_{0}=0$; Fig A(B)), *P* preys on *F_0_* will show more stable regions of species coexistence ($\theta_{0}\neq0$; Fig A(D)). Similarly, predation of *F_0_* by *P* contributes to community stability and is also presented in other food web structures (Fig A(A) and Fig A(C)). Specifically, compared to *P* does not prey on *F_0_* and *C* ($\theta_{C}=0, \theta_{0}=0$; Fig A(C)), *P* preys on *F_0_* but not *C* ($\theta_{0}\neq0, \theta_{C}=0$) may promote community stability (u1<0.15 and β<0.1; Fig A(A)).


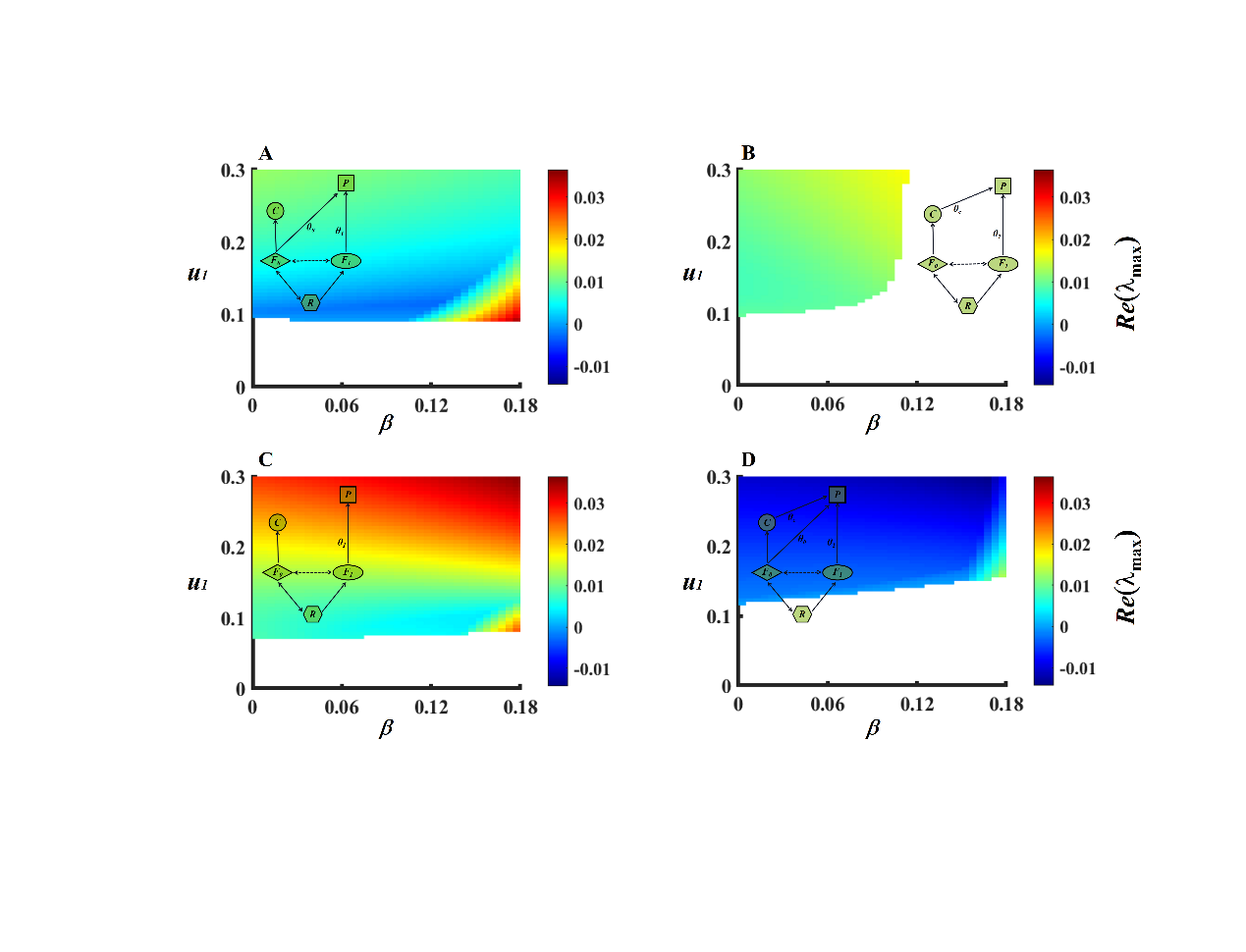


**Fig A.** Network structures and local stability change with the interspecific competition (*β*) and consumption rate (*u_1_*) in the resource-mutualist-exploiter-predator communities. Stable coexistence of species is achieved when *Re*(*λ_max_*)<0. In each simulation case, the blue regions have higher stability than the red regions, and the empty white regions denote no solution to network modules. Key parameters of the four-species model: g=0.1, *u_1_*$\in$[0, 0.3], *β_10_=β_01_*=*β*$\in$[0, 0.18] and other parameter values: r1=1, k=0.2, m=0.4, v=0.3, ac=0.2, u0=0.2, uc=0.25, d0=0.1, d1=0.1, dc=0.1, dp=0.1, $\alpha_{0}$=0.1, $\alpha_{1}$=0.1, $\alpha_{C}$=0.1, $\alpha_{P}$=0.1, ec=1, ep=1.

However, above theoretical predictions are contrary to the results in the main text (Fig 3), which asserts that P preying on F0 will be detrimental to the stability of mutualistic systems. The view that P not preying on F0 will be beneficial to the stability of mutualism has been supported by early empirical work (Wang et al. 2014), which found that predation of the ant (P) indirectly reinforces mutualism through suppressing parasitic infestation, thereby enhancing reproductive success in both the mutualist (F0) and fig tree (R). An important reason for the inconsistency in the predictions of these two models is whether the life history of the fig tree (i.e., the basal resource R) is taken into account in the model, that is, the life cycle of fig trees is much longer than that of fig wasps (i.e., F0, F1, C) and the top predator (i.e., P). Compared with directly adding the basal resource to the model (Fig A), it is more reasonable to ignore the resources and regard the mutualist as one potential resource (Fig 3 in main text). Therefore, we finally chose the mutualist-exploiter-specialist predator-top predator (MEST) community as the research system.

**References**

Addicott, J.F. (1981). Stability properties of two-species models of mutualism: simulation studies. Oecologia, 49, 42–49.

May, R.M. (1981). Models for two interacting populations. In: Theoretical Ecology: Principles and Applications (ed. May, R.M.). Blackwell Scientiﬁc, Oxford, UK, pp. 49–70.

Wang, B., Geng, X.Z., Ma, L.B., Cook, J.M., Wang, R.W. (2014) A trophic cascade induced by predatory ants in a fig-fig wasp mutualism. J Anim Ecol., 3(5):1149-57.

Wolin, C.L. & Lawlor, L.R. (1984). Models of facultative mutualism: density effects. Am. Nat., 124, 843–862.
