# Supplementary material for: Evolution of foraging behaviour induces variable complexity-stability relationships in mutualist-exploiter-predator communities
Source: PLoS Comput Biol. 2025 Jul 9;21(7):e1013245. doi: 10.1371/journal.pcbi.1013245 (PMC12240360; doi:10.1371/journal.pcbi.1013245)
Supplement: S4 Appendix — (DOCX) [file pcbi.1013245.s004.docx]

**S4 Appendix---Population chaos and flexible network structures in a complex community**

Similar to chaotic dynamics presented in the four-species MEST community (Fig 2 in the main text), our theoretical results suggest that each species may exhibit oscillatory coexistence in a specific MEST community with a given combination of parameters (*N*=9; Fig A). In Fig A, both population biomass (Fig A(A)) and foraging efforts (Fig A(B)) show chaotic dynamics; moreover, the network structure is flexible and changes over time. For instance, at *t* = 40,000, two foraging efforts (blue ellipses) become extinct (red dashed line; Fig A(C)), whereas only one foraging effort goes extinct (red dashed line; Fig A(D)) when *t* = 50,000; moreover, the intensity of foraging efforts (thickness of black solid lines) also changes over time (Fig A(C) and Fig A(D)).





**Fig A.** Population chaos and flexible network structures in a specific MEST community (*N*=9). (A) irregular oscillations of population biomass; (B) irregular oscillations of foraging efforts; (C, D) network structures change with time. Key parameters of the nine-species model: *r_1_*=0.3497, *r_2_*=0.3473, *r_3_*=0.3649, *r_4_*=0.3474, *r_5_*=0.3500, *r_6_*=0.3617; *d_i_*=0.05; *u_i_*=0.3; *g*=0.28; *β_i0_=β_ji_*=*β*=0.09, and other parameter values are presented in Table 1. The initial value is (*F_0_*=0.5, *F_1_*=0.2696, *F_2_*=0.2805, *F_3_*=0.2818, *F_4_*=0.3105, *F_5_*=0.2965, *F_6_*=0.2871, *C*=0.2, *P*=0.1, *θ_0_*=0.6, *θ_i_*=0.2/6.
