# Supplementary material for: Evolution of foraging behaviour induces variable complexity-stability relationships in mutualist-exploiter-predator communities
Source: PLoS Comput Biol. 2025 Jul 9;21(7):e1013245. doi: 10.1371/journal.pcbi.1013245 (PMC12240360; doi:10.1371/journal.pcbi.1013245)
Supplement: S5 Appendix — (DOCX) [file pcbi.1013245.s005.docx]

**S5 Appendix---Model fitting based on empirical data**

**Summary**

**Freshwater ecosystems**

|  | Equation | P-value | R^2^ |
| --- | --- | --- | --- |
| **Linear fit** | **y=-1.05279x+0.89125** | **0.04981** | **0.24768** |
| **Nonlinear curve fit (Parabola)** | y=-2.57739x^2^+1.89848x+0.09896 | 0.12973 | 0.26963 |
| **Nonlinear curve fit (Cubic)** | y=23.98984x^3^-44.18109x^2^+25.10233x-4.03736 | 0.22795 | 0.29339 |

**Marine ecosystems**

|  | Equation | P-value | R^2^ |
| --- | --- | --- | --- |
| **Linear fit** | y=-0.03321x+0.17286 | 0.95182 | 2.08493E-4 |
| **Nonlinear curve fit (Parabola)** | **y=-16.60223x^2^+16.04436x-3.54895** | **0.04738** | **0.30146** |
| **Nonlinear curve fit (Cubic)** | y=-72.79215x^3^+86.88784x^2^-31.80847+3.64224 | 0.0823 | 0.33399 |
