# Supplementary material for: Evolution of foraging behaviour induces variable complexity-stability relationships in mutualist-exploiter-predator communities
Source: PLoS Comput Biol. 2025 Jul 9;21(7):e1013245. doi: 10.1371/journal.pcbi.1013245 (PMC12240360; doi:10.1371/journal.pcbi.1013245)
Supplement: S6 Appendix — (ZIP) [file pcbi.1013245.s006.zip › S6_Appendix/S6_Appendix.docx]

**S6 Appendix---Simulation codes for all figures**

Regarding Fig 2（Fig A in **S4 Appendix** adopts the same methodology）

(1) Execute the code bifur_r.m to obtain Fig 2A;

(2) Execute the code run_lyapunov.m to obtain Fig 2B;

(3) Execute the code runwanglin.m to obtain Fig 2C and Fig 2D.

Regarding Fig 3（Fig A in **S3 Appendix** adopts the same methodology）

(1) Execute the code equilibrium.m to compute all equilibrium points of the equation system, classify these equilibrium points according to different network structures, and store the results in equal_0001;

(2) Execute the code jacob.m to calculate the Jacobian matrix at each equilibrium point, compute the eigenvalues, extract the maximum real part of the eigenvalues, and generate Fig 3 based on these results.

Regarding Fig 4

(1) Access the differential equation solver implemented in adapt.m;

(2) Execute numerical simulations using newrun.m to determine species survival counts across varying g and β values;

(3) Generate graphical outputs via plot.m, with each parameter-specific subplot saved as individual .fig files (following the naming convention: [sequence]figure[figure#]beta[β-value]g[g-value].fig), then composite these 12 subplots to form Fig 4.

Regarding Fig 5

Execute the script figure5final_empirical.m to generate scatter plots based on freshwater and marine data, perform curve fitting, and produce the latter two subplots of Fig 5.
