# Supplementary material for: Evolution of foraging behaviour induces variable complexity-stability relationships in mutualist-exploiter-predator communities
Source: PLoS Comput Biol. 2025 Jul 9;21(7):e1013245. doi: 10.1371/journal.pcbi.1013245 (PMC12240360; doi:10.1371/journal.pcbi.1013245)
Supplement: Attachment — Submitted filename: Response to Reviewers.docx [file pcbi.1013245.s008.docx]

**List of Responses**

Dear Editor and Reviewers:

Thank you for your comments related to our manuscript entitled “Evolution of foraging behaviour induces variable complexity-stability relationships in a multiplex community with multiple interaction types”. (Ms. Ref. No.: PCOMPBIOL-D-24-01756). Those comments are all valuable and very helpful for revising and improving our paper, as well as an important guiding significance to our researches. We have studied the comments carefully and have edited the manuscript to meet with the comments. Revised parts are marked in red in the paper.

The main corrections in the paper and the responds to the comments of both editor and reviewers are as following:

**Responds to the editor’s comments:**

**1. Response to comment**: *Thank you for your submission. The manuscript has received mixed feedback from three reviewers. While all three recognize the novelty and potential impact of addressing complexity-stability relationships in ecological networks with adaptive foraging, they highlight critical issues that must be addressed. Reviewer 1 and Reviewer 3 recommend major revisions, focusing on improving model justification, clarity of results, and empirical validation. However, Reviewer 2 raises stronger concerns, including the lack of clear research questions, methodological ambiguity, and ecological relevance, and recommends rejection.*

*I think that the manuscript could make a significant contribution to the field if these issues are **thoroughly** resolved.*

*Therefore, I am recommending a major revision supported by point-by-point responses to the reviewers' comments.*

*In particular,*

** Reviewer 1 questions the generalizability of the findings due to the simplistic model structure and asks for clarification on its applicability to larger ecosystems, requests stronger justification for parameter values and suggests robustness analyses (also shared by other reviewers), and criticizes the inconsistency in how stability is measured, noting the shift from eigenvalue-based analysis to community persistence in larger networks. They also recommend improving the clarity of terms, figure labeling, and technical definitions.*

** Reviewer 2 criticises the lack of clear objectives and hypotheses, unclear / unjustified methodological details, and most importantly, highlights issues with the model: exclusion of basal resources, decoupling of competition dynamics, and parameter sensitivity (again). They also note that empirical validation with freshwater and marine systems is poorly integrated and inadequately explained (again, shared by Rev 3).*

*Reviewer 3 criticises the lack of clarity in linking different model findings and suggests additional simulations to control of confounding factors (so the same theme of parameter and structural sensitivity and robustness analysis again), and asks for more rigorous statistical approaches in the empirical validation and better explanation of the modification of connectance. They also highlight (and again a common theme) difficulties in following the choice of parameter variation and understanding which modeling elements drive specific results, and suggest enhancing figure clarity and consistency to aid interpretation.*

*Overall, therefore I suggest that your revision addresses the following in particular:*

**Response**: Once again, thank you for giving us the opportunity to revise the manuscript and for your assistance in summarizing the feedback from the three reviewers. In the subsequent sections, we have addressed each comment point by point.

**2. Response to comment**: ** Clearly state the research question(s) and hypotheses in the introduction (Reviewer 2).*

**Response**: Thank you for your comments. We have refined research questions of this study, and restructured the descriptions in the Introduction section. These modifications are reflected in Lines 105-157.

**3. Response to comment**: ** Clarify/justify key assumptions and such as parameter values, and provide robustness or sensitivity analyses (Reviewers 1 & 2).*

**Response**: Thank you for your comments. We have conducted sensitivity analyses on model parameters (Appendix B in the supplementary material), with detailed descriptions incorporated into the main text. These modifications are reflected in Lines 238, 318-321, and 750-751.

**4. Response to comment**: ** Address model limitations (structural sensitivity), by incorporating basal resources, or justify their exclusion analytically.*

**Response**: Thank you for your comments. We have conducted sensitivity analyses on model structure (Appendix C in the supplementary material), with detailed descriptions incorporated into the main text. These modifications are reflected in Lines 171-174, 238-239, 464-473, and 752-753.

**5. Response to comment**: ** Explain the treatment of competition dynamics and analyse the coupling of intra- and interspecific competition (Reviewer 2).*

**Response**: Thank you for your comments.

Our previous work demonstrated that interspecific interference competition among fig wasps can reduce the virulence of fig wasps and stabilize the fig-wasp mutualism (Wang et al. 2009). In the current study, we fixed the intraspecific competition intensity (α_0_ = 0.13) and analyzed changes in community stability under three distinct interspecific competition scenarios: low value (β = 0.1), intermediate value (β = 0.14), and high value (β = 0.16). These modifications are reflected in Lines 335-336, 344-345, and 349-350.

**References:**

Wang RW, Ridley J, Sun BF, Zheng Q, Dunn DW, et al. Interference Competition and High Temperatures Reduce the Virulence of Fig Wasps and Stabilize a Fig-Wasp Mutualism. PLOS ONE, 2009, 4(11): e7802.

**6. Response to comment**: ** Provide detailed descriptions of empirical model fitting and comparison, and clearly explain the relationship between model predictions and observed data (Reviewers 2 & 3).*

**Response**: Thank you for your comments. We have conducted empirical model fitting (Appendix E in the supplementary material), with detailed descriptions incorporated into the main text (see Lines 259-260, 357-361, 366-368, 373-375, 379-381, 493-495, and 756).

**7. Response to comment**: ** Reconcile the use of different stability metrics and justify their selection for specific network sizes (Reviewer 1).*

**Response**: Thank you for your comments. In the “Model analysis” section, we have clarified the reason that distinct stability metrics were applied to the four-species MEST (i.e., Fig. 3) and general MEST (i.e., Fig.4) systems. These modifications are reflected in Lines 240-246.

In addition, the selection of the specific network size (N = 9) was designed to align with the chaotic dynamics observed in the four-species MEST community (Fig. 2), highlighting the potential prevalence of population chaos within the adaptive network framework of this study. This discussion has been relocated to the Supplementary Materials (Appendix D), with supporting explanations provided in Lines 258, 431-433, and 754-755.

**8. Response to comment**: ** Enhance figure consistency (e.g., color schemes) and labeling for clarity, and reorganize the manuscript to place the methods section earlier (Reviewers 1 & 3).*

**Response**: Thank you for your comments. We have revised and supplemented key figures in the main text: first, chaotic dynamics of the four-species MEST community have been incorporated into Fig. 2, with corresponding descriptions in Lines 229-232 and 266-289; second, the color labels in Fig. 3 have been standardized and minor layout adjustments have been made to Fig. 4; Finally, Fig. 5 and Fig. 6 have been reordered (see Lines 356-411), and the caption of Fig. 5 has been expanded to include additional explanatory notes (see Lines 366-368).

In addition, we have placed the methods section earlier, which presented in Lines 159-262.

**9. Response to comment**: ** Strengthen the connection between model dynamics (e.g., feedback loops) and observed stability trends through additional analyses or simulations (Reviewer 3).*

**Response**: Thank you for your comments.

In this study, we propose a potential qualitative mechanism underlying the complexity-stability relationships revealed in Fig. 4, that is, adaptive foraging by the top predator can modulate system stability by altering the dominant positive/negative feedback loops within the community (trait-mediated indirect effects), as elaborated in Lines 396-399, 425-427, and 515-518.

Furthermore, we delve deeper into the intrinsic linkages among adaptive foraging, positive-negative feedbacks, and multimodal complexity-stability relationships (see Lines 518-532).

Finally, we analyze the similar trends between model predictions and empirical data analyses in Fig. 5 and Appendix E in the supplementary material (see Lines 366-368, 373-375, and 379-381).

**10. Response to comment**: *If you do choose to undertake the revision, the final decision will depend on the point-by-point responses, revisions, and potentially, further review (ideally by the same reviewers).*

**Response**: Thank you very much.

**11. Response to comment**: *Journal Requirements: 1) We noticed that you used the phrase 'not shown' in the manuscript. We do not allow these references, as the PLOS data access policy requires that all data be either published with the manuscript or made available in a publicly accessible database. Please amend the supplementary material to include the referenced data or remove the references. 2) We have noticed that you have uploaded Supporting Information files, but you have not included a list of legends. Please add a full list of legends for your Supporting Information files after the references list.*

**Response**: Thank you for your comments. We have deleted “(not shown here)” presented Line 412; moreover, we have re-organized the supplementary material by including Appendices A-F (See Lines 748-757).

**12. Response to comment**: *3) Please ensure that the funders and grant numbers match between the Financial Disclosure field and the Funding Information tab in your submission form. Note that the funders must be provided in the same order in both places as well." Currently, the order of these grants "32201264" ,32271548" and "U2102221" is different in both places.*

*Please indicate by return email the full and correct funding information for your study and confirm the order in which funding contributions should appear. Please be sure to indicate whether the funders played any role in the study design, data collection and analysis, decision to publish, or preparation of the manuscript.*

**Response**: Thank you for your comments. We have added “Funding” section presented in Lines 557-564.

**Thank you very much for your valuable comments and suggestions.**

**Responds to the reviewer’s comments:**

**Reviewer #1: Comments**

**1. Response to comment**: *This manuscript explores how adaptive foraging behavior in complex ecological networks influences community stability in systems with both mutualistic and antagonistic interactions. Using an adaptive network model, the authors demonstrate that changes in foraging preferences can result in diverse stability patterns, including single and double peaks, potentially explaining the varying stability trends observed in real-world ecosystems, such as freshwater and marine environments. The proposed adaptive framework holds promise for capturing both observed patterns and differences in the complexity-stability relationships across these systems.*

*This manuscript offers a novel theoretical framework with valuable insights into the complexity-stability debate, which has the potential to make a meaningful contribution to the field. However, there are several areas where significant revisions are needed. In particular, I believe the authors should provide stronger justification for their model assumptions, clarify the treatment of stability metrics, and strengthen the connections to empirical systems. I outline three major concerns along with a list of minor issues, which I hope the authors will find constructive in improving the manuscript.*

**Response**: Thank you very much for your comments. We have addressed each of your valuable comments point by point.

**2. Response to comment**: *Major Issues*

*1. Simplistic Community Structure:*

*The manuscript focuses on a simple omnivorous food web incorporating mutualistic and antagonistic relationships with only four species across three trophic levels: top predator, specialist predator, mutualist, and exploiter. While such a setup is useful for tractability and analysis, it raises questions about the generalizability of the findings to real-world ecosystems. Specifically, what would happen if there were more than one specialist predator or more layers of interactions? The authors need to justify how their framework applies to larger, more realistic ecological systems.*

**Response**: Thank you for your suggestions.

First, our analytical framework is grounded in empirical ecological networks (i.e., the fig-fig wasp system in Fig. 1A; Wang et al. 2014; Wang et al. 2020).

Second, we justify the exclusion of the basal resource (i.e., fig tree) in this adaptive network model through both qualitative arguments (i.e., the markedly longer life cycle of the fig tree compared to fig wasps and the predatory ant) and quantitative validation (structural sensitivity analyses; Appendix C), our adaptive network framework treats the mutualist as one potential resource (see Lines 171-174, 238-239, 464-473, and 752-753).

Third, simulation analyses revealed that increasing interspecific competition intensity may shift the complexity-stability relationship from a monotonically decreasing pattern to a unimodal pattern (initially increasing then decreasing). These predictions align well with observed complexity-stability relationships in freshwater communities (characterized by low salinity and weak competition) versus marine communities (exhibiting high salinity and intense competition), as demonstrated in Fig. 5 (see Lines 357-361, 366-368, 373-375, 379-381, and Appendix E).

Finally, in the “Future direction” section, we emphasize the critical need to integrate multispecies interactions and multilayer relationships within the current framework (see Lines 539-542).

**References**

Wang B, Geng XZ, Ma LB, Cook JM, Wang RW. A trophic cascade induced by predatory ants in a fig–fig wasp mutualism. Journal of Animal Ecology. 2014;83(5):1149-57.

Wang L, Liu Y-P, Wang R-W. Weak predation strength promotes stable coexistence of predators and prey in the same chain and across chains. International Journal of Bifurcation and Chaos. 2020;30(15):2050228.

**3. Response to comment**: *Additionally, it is unclear how this model relates to the two empirical systems—freshwater and marine—mentioned in the paper. The primary difference between the two empirical communities is the intensity of interspecific competition, which is deduced in this manuscript. This difference leads to variations in adaptive responses and ultimately drives the contrasting stability outcomes between freshwater and marine systems. However, this difference requires further justification.*

**Response**: Thank you for your suggestions.

Simulation analyses revealed that increasing interspecific competition intensity may shift the complexity-stability relationship from a monotonically decreasing pattern to a unimodal pattern (initially increasing then decreasing). These predictions align well with observed complexity-stability relationships in freshwater communities (characterized by low salinity and weak competition) versus marine communities (exhibiting high salinity and intense competition), as demonstrated in Fig. 5 (see Lines 357-361, 366-368, 373-375, 379-381, and Appendix E).

**4. Response to comment**: *2. Parameter Values:*

*The parameter values in Table 1 lack adequate justification. For instance:*

*- Why is the growth rate of F0 set at 0.5? Is this value based on empirical systems? If so, the authors should specify which system. If not, a robustness analysis testing other growth rate values would be necessary.*

*- Why are the growth rates of Fi always smaller than those of F0? This assumption should be explained, particularly in the context of ecological realism.*

*- The conversion efficiency is set at 1, implying perfect conversion. This is an idealized assumption that may not hold in real-world systems. The authors should clarify why this assumption was made and discuss its potential impact if it does not hold.*

*- The density-dependent coefficients (alpha0, alphaC, alphaP) are set at 0.1, 0.12, and 0.13, respectively, but there is no clear reasoning for these specific values. The manuscript would benefit from an explanation of why these coefficients vary in this way and how sensitive the model outcomes are to changes in these parameters.*

*- The intensity of foraging adaptation and competition parameters also require clearer biological or ecological justification. Without this, it is difficult to assess the model’s applicability to real systems.*

**Response**: Thank you for your suggestions.

In the adaptive network model, all model parameters (Table 1) can be chosen based on the stability criteria (Temam 2012) under the non-negative equilibrium. We calculate a positive equilibrium of species coexistence and analyze the maximal real part of all eigenvalues (Re(λmax)) under the equilibrium, and obtain a stable state when Re(λmax) < 0. These modifications are reflected in Lines 220-224.

We have conducted sensitivity analyses on model parameters (Appendix B in the supplementary material), with detailed descriptions incorporated into the main text. These modifications are reflected in Lines 238, 318-321, and 750-751.

**References**

Temam R. Infinite-dimensional dynamical systems in mechanics and physics: Springer Science & Business Media, 2012.

**5. Response to comment**: *3. Inconsistency in Stability Metrics:*

*There is a fundamental inconsistency in how stability is defined and measured in the manuscript. For small networks, the authors use local stability analysis based on eigenvalues, which measures how quickly a system returns to equilibrium after perturbations. This is a classic and well-established approach.*

*For larger networks, however, the authors shift to using community persistence (the fraction of remaining species after long simulations) as a proxy for stability. This measure is more aligned with species diversity rather than stability, as it does not capture dynamic responses to perturbations. Given the long-standing debate on the diversity-stability relationship, extreme care must be taken when using diversity-related measures as a proxy for stability.*

*The manuscript should clearly differentiate between these two metrics and their implications. Unless additional analyses are performed to measure stability in larger communities (e.g., analyzing eigenvalues or resilience metrics), the claims about stability in large networks are not fully convincing.*

**Response**: Thank you for your suggestions.

In the “Model analysis” section, we have clarified the reason that distinct stability metrics were applied to the four-species MEST (i.e., Fig. 3) and general MEST (i.e., Fig.4) systems. These modifications are reflected in Lines 240-246.

In addition, for larger networks (general MEST communities), we used the community persistence (e.g., network robustness changes with the connectance; Wang et al. 2023) as a proxy for stability.

**References**

Wang L, Li J, Wang T, Liao J. A positive complexity-stability relationship emerges in pollinator-plant-consumer tripartite networks disturbed by plant invasion[J]. Ecological Modelling, 2023, 484: 110461.

**6. Response to comment**: *Minor Issues*

*1. Some terms, such as “positive-negative feedback,” could be simplified or better explained.*

**Response**: Thank you for your suggestions.

In this study, the positive-negative feedback denotes maximal feedback loops $\left( i.e., P\underset{\to}{+}AF\underset{\to}{+}C\underset{\to}{-}F_{0}\underset{\to}{-}F_{1}\underset{\to}{+}P \& P\underset{\to}{+}AF\underset{\to}{+}F_{1}\underset{\to}{-}F_{0}\underset{\to}{+}C\underset{\to}{+}P \right)$ include both positive ($P\underset{\to}{+}AF\underset{\to}{+}C\underset{\to}{-}F_{0}\underset{\to}{-}F_{1}\underset{\to}{+}P$) and negative ($P\underset{\to}{+}AF\underset{\to}{+}F_{1}\underset{\to}{-}F_{0}\underset{\to}{+}C\underset{\to}{+}P$) feedback loops, that is, “positive-negative feedback” means “positive and negative feedbacks”, and these modifications are reflected in Lines 406, 408, 412, and 529.

**7. Response to comment**: *2. Figures, particularly Figures 2 and 3, would benefit from consistent labeling and coloring for stability regions to make the results easier to interpret.*

**Response**: Thank you for your suggestions.

We have revised and supplemented key figures in the main text: first, chaotic dynamics of the four-species MEST community have been incorporated into Fig. 2, with corresponding descriptions in Lines 229-232 and 266-289; second, the color labels in Fig. 3 have been standardized and minor layout adjustments have been made to Fig. 4.

**8. Response to comment**: *3. Including brief definitions for technical terms like "trait-mediated indirect effects" and "Holling type I functional response" would help readers less familiar with ecological modeling.*

**Response**: Thank you for your suggestions.

For trait-mediated indirect effects, we added “(i.e., foraging preference trait of the top predator modulates the system stability by altering the dominant positive or/and negative feedback loops)” presented in Lines 397-399; for Holling type I functional response, we added “or linear function” presented in Line 187.

**9. Response to comment**: *4. In line 456, the equation mentions "consumption of C" instead of F0. This seems inconsistent with the context and should be clarified.*

**Response**: Thank you for your suggestions. We have changed “*C*” to “*F_0_*” presented in Line 193.

**10. Response to comment**: *5. While the GitHub code link is provided, a README file with instructions on how to run the code would make it more accessible to researchers who want to replicate or build upon the study.*

**Response**: Thank you for your suggestions. We have reuploaded the simulation code (MATLAB).

**Thank you very much for your valuable comments and suggestions.**

**Reviewer #2: Comments**

**1. Response to comment**: *In the current study entitled “Evolution of foraging behaviour induces variable complexity-stability relationships in a multiplex community with multiple interaction types”, the authors, Wang et al. extend from their previous work to create a multi-species (i.e., beyond four species) version the MEST model, which includes a top predator that feeds on all others, a specialist predator that feeds on the mutualist, as well as a mutualist and one or more exploiters that feed on a shared resource. The goal of the study is to explore the influence of foraging preference evolution (in terms of the top predator’s foraging effort allocation among prey species) on the system’s stability (in terms of local stability, evaluated by eigen-value analysis of the Jacobian, and community persistence, in terms of the number of species that remain after a long run) when the system has both antagonistic and mutualistic interactions. This research direction is inheriting, and combining the topics, explored by M. Kondoh (ref 13 and 29 in the manuscript).*

*While the study indeed targets an existing gap in the field of complexity-stability debate (i.e., the effect of adaptive foraging with mix-type interactions), and the reported behaviours of the model (especially the uni- and bi-modality in the complexity-stability relationships) are somewhat interesting, unfortunately, I find the manuscript in its current form rather unsatisfying, and this is due to a combination of the arbitrariness of the modelling part, the ambiguity of the overall methodological description, and thus, the unconvincing discoveries and relevant ecological interpretation. Below I elucidate my major concerns, and I hope this could help the authors to improve the study.*

**Response**: Thank you very much for your valuable comments. We have addressed each of your valuable comments point by point.

**2. Response to comment**: *1. There is no clear research question nor hypotheses stated, and therefore the readership would not know what to expect in this manuscript even after reading the introduction---is the current study going to manipulate network structure, the composition of antagonistic vs. mutualistic elements, interaction strengths, or other key parameters? What is going to be “tested” after all? The journal PLOS Computational Biology emphasises reliability and significance of biological discovery through computation, so a sound framing of the research question, and biologically relevant hypotheses, are definitely needed.*

**Response**: Thank you for your valuable comments.

We have refined research questions of this study, and restructured the descriptions in the Introduction section. These modifications are reflected in Lines 105-157.

**3. Response to comment**: *2. Following the previous point, some of such information are only given in the methods section, however without reasoning or justification, and relevant details are only very loosely described. So, even after reading the methods, the readership would not know why the authors decided to test such aspects of the model, and why manipulated in such ways. Some examples are:*

*2-1. There is no notice nor reasoning for that the authors are testing 4-sp and multi-sp models separately. Are these two parts answering different questions? What is the goal for extending 4-sp to multi-sp? None of these are explained.*

**Response**: Thank you for your valuable comments.

In the “Model analysis” section, we have clarified the reason that distinct stability metrics were applied to the four-species MEST (i.e., Fig. 3) and general MEST (i.e., Fig.4) systems. These modifications are reflected in Lines 240-246.

In addition, the connection between the four-species MEST community (Figs. 2-3) and general MEST communities (Fig. 4 and Fig. S11) is manifested in three aspects:

(i) Parameter consistency: most parameters in the general MEST model align with those in the four-species MEST system;

(ii) Stability comparison under adaptation strengths: Fig. 2 illustrates changes in system stability (persistence or robustness) under low (g=0.08), intermediate (g=0.28), and high (g=0.48) adaptation strengths for the four-species system, while Fig. 4 similarly analyzes stability variations in the general system under these three adaptation strengths;

(iii) Chaotic dynamics: both the simplified system (Fig. 2) and the complex system (Fig. S11) exhibit chaos under the adaptive dynamics framework.

**4. Response to comment**: *2-2. When moving to the multi-sp model, why it is only the number of exploiters being increased, but not the other roles? Is it for mimicking a realistic system (e.g., the wasp system in Fig. 1A)? But if so, is the goal of the study to specifically address the dynamics in that system, or to tackle a very general mechanism in ecological dynamic systems? From the introduction it sounds like the latter; yet from the methods, and the fact that the authors associate the findings to freshwater and marine food webs, not so.*

**Response**: Thank you for your valuable comments.

We apologize for not clarifying the logical flow of the entire text and have reorganized it as follows:

First, our analytical framework is grounded in empirical ecological networks (i.e., the fig-fig wasp system in Fig. 1A; Wang et al. 2014; Wang et al. 2020).

Second, we justify the exclusion of the basal resource (i.e., fig tree) in this adaptive network model through both qualitative arguments (i.e., the markedly longer life cycle of the fig tree compared to fig wasps and the predatory ant) and quantitative validation (structural sensitivity analyses; Appendix C), our adaptive network framework treats the mutualist as one potential resource (see Lines 171-174, 238-239, 464-473, and 752-753).

Third, simulation analyses revealed that increasing interspecific competition intensity may shift the complexity-stability relationship from a monotonically decreasing pattern to a unimodal pattern (initially increasing then decreasing). These predictions align well with observed complexity-stability relationships in freshwater communities (characterized by low salinity and weak competition) versus marine communities (exhibiting high salinity and intense competition), as demonstrated in Fig. 5 (see Lines 357-361, 366-368, 373-375, 379-381, and Appendix E).

Finally, in the “Future direction” section, we emphasize the critical need to integrate multispecies interactions and multilayer relationships within the current framework (see Lines 539-542).

**References**

Wang B, Geng XZ, Ma LB, Cook JM, Wang RW. A trophic cascade induced by predatory ants in a fig–fig wasp mutualism. Journal of Animal Ecology. 2014;83(5):1149-57.

Wang L, Liu Y-P, Wang R-W. Weak predation strength promotes stable coexistence of predators and prey in the same chain and across chains. International Journal of Bifurcation and Chaos. 2020;30(15):2050228.

**5. Response to comment**: *2-3. And, speaking of the empirical validation with freshwater and marine food webs, this part is not mentioned or explained anywhere at all. It just appears out of the blue in the results, without any context. What models are those fitted against the data? What are the alternative models and are there any model selection process? Wouldn’t a unimodal model also (maybe better) fit to the data of Fig. 6C? Having this bit in the manuscript does not help make the model more convincing, instead incur confusions.*

**Response**: Thank you for your important comments.

Simulation analyses revealed that increasing interspecific competition intensity may shift the complexity-stability relationship from a monotonically decreasing pattern to a unimodal pattern (initially increasing then decreasing). These predictions align well with observed complexity-stability relationships in freshwater communities (characterized by low salinity and weak competition) versus marine communities (exhibiting high salinity and intense competition), as demonstrated in Fig. 5 (see Lines 357-361, 366-368, 373-375, 379-381, and Appendix E).

**6. Response to comment**: *2-4. L495: the description here itself does not give any detail regarding how the authors derive the equilibrium of a dynamic system with adaptively changing interaction strengths. And thus, it is unclear at what states the eigenvalues are evaluated, and how such states are derived/approached.*

**Response**: Thank you for your comments. We have reorganized the descriptions presented in Lines 240-241.

**7. Response to comment**: *2-5. L498 onwards: the authors state that “it is more difficult…” so that they use an alternative way (remaining sp after a long run) of quantifying stability in the multi-sp model other than the eigenvalue. I do not quite get this reasoning. What are the boundary equilibrium points, and why is it difficult to list all possible network structures? How large is the network mentioned here after all---it is not mentioned; I can only tell from Fig. 3 that the authors simulated network sizes from 5 to 35 (which, again, is a setting not mentioned nor justified elsewhere), yet 35 does not seem like a very big network. I do not get why the approach doable to a 4-sp system (which, as pointed out in the previous point, is not explained) cannot be applied to a 35-sp system, especially when all the species added to the system are of the same role (exploiters) so the complexity is actually constrained.*

**Response**: Thank you for your suggestions.

For the 35-species community, the network structure will change when the top predator selectively preys on the specialist predator and 32 exploiters, so the total number of boundary equilibrium points is 2^33^-2 (i.e., $C_{33}^{1}+C_{33}^{2}+\cdots+C_{33}^{32}=2^{33}-2$), which is more than 8.5899🞨10^9^. We have reorganized the descriptions presented in Lines 242-246.

**8. Response to comment**: *2-6. L513: Why highlighting specifically a N = 9 case? Is this size specifically representative or ecologically relevant? Why not other sizes?*

**Response**: Thank you for your suggestions.

The selection of the specific network size (N = 9) was designed to align with the chaotic dynamics observed in the four-species MEST community (Fig. 2), highlighting the potential prevalence of population chaos within the adaptive network framework of this study. This discussion has been relocated to the Supplementary Materials (Appendix D), with supporting explanations provided in Lines 258, 431-433, and 754-755.

**9. Response to comment**: *2-7. Why the main focus is on beta, g, and N, but not other aspects/parameters? What’s the rationale of this decision? And how the investigation is carried out---e.g., in terms of the range of values that the authors explore?*

**Response**: Thank you for your suggestions.

In this study, to gain a deeper understanding of the effects of key parameters on the stability of simplified systems (N=4, Fig. 2 and Fig. 3) and complex systems (N=5-35; Fig. 4), we fixed most parameters and focused on analyzing how critical parameters regulate system stability (i.e., local stability, community persistence or robustness): in Fig. 2, we fixed the network size (N=4) and interspecific competition (β=0.18) while varying the adaptation strength (g); in Fig. 3, we fixed the adaptation strength (g=0.28) and network size (N=4) while varying the interspecific competition (g); in Fig. 4, we simultaneously varied the network size (N), interspecific competition (β), and adaptation strength (g); in addition, for the simplified system (N=4; Figs. 2-3), we conducted sensitivity analyses of each model parameter to further explore its dynamic behaviour (Table S1 in Appendix B).

**10. Response to comment**: *2-8. Table 1: How are these values determined, and what are the units of them? Without any justification, I can hardly believe that these values are within a biologically meaningful or realistic range. Also, seemingly there is no sensitivity analyses on the simulations at all? If all the conclusions are drawn from only one set of parameters, wouldn’t all the discovered patterns are in fact outcomes that are sensitive to the parameter chosen, rather than that of general ecological meaning?*

**Response**: Thank you for your suggestions.

In the adaptive network model, all model parameters (Table 1) can be chosen based on the stability criteria (Temam 2012) under the non-negative equilibrium. We calculate a positive equilibrium of species coexistence and analyze the maximal real part of all eigenvalues (Re(λmax)) under the equilibrium, and obtain a stable state when Re(λmax) < 0. These modifications are reflected in Lines 220-224.

Similar to our early theoretical work in the fig-wasp system (Wang et al. 2020a; Wang et al. 2020b), the unit of each parameter is dimensionless. These modifications are reflected in Lines 224-225.

We have conducted sensitivity analyses on model parameters (Appendix B in the supplementary material), with detailed descriptions incorporated into the main text. These modifications are reflected in Lines 238, 318-321, and 750-751.

**References**

Temam R. Infinite-dimensional dynamical systems in mechanics and physics: Springer Science & Business Media, 2012.

Wang L, Liu Y-P, Wang R-W. Weak predation strength promotes stable coexistence of predators and prey in the same chain and across chains. International Journal of Bifurcation and Chaos. 2020a, 30(15):2050228.

Wang L, Yang L-Y, Zhang F, Wang R-W. Moderate parasitoidism on pollinators contributes to population oscillations and increases species diversity in the fig-fig wasp community. Theoretical Ecology. 2020b, 13(2):251-60.

**11. Response to comment**: *3. My biggest concern lies on the model itself.*

*3-1. Why not including the basal resource, as indicated in Fig. 1A, as a state variable into the model? Without this entity, the model ignores several important feedbacks in the system. For instance, the mutualist in the model in effect is not a “mutualist” at all in the current system---there is no other entity that is “mutually benefitting” (+/+) with it. As illustrated in Fig. 5A, the interactions between the mutualist and the exploiters are effectively competition (-/-) and are incorporated into the model’s interaction matrix just as all other interactions; however, these are actually indirect interaction and should be mediated through the abundance of a basal resource, but such resource-dependency (which would be a constraining coupling between direct and indirect interaction strengths) is excluded from the model.*

**Response**: Thank you for valuable comments.

We justify the exclusion of the basal resource (i.e., fig tree) in this adaptive network model through both qualitative arguments (i.e., the markedly longer life cycle of the fig tree compared to fig wasps and the predatory ant) and quantitative validation (structural sensitivity analyses; Appendix C), our adaptive network framework treats the mutualist as one potential resource (see Lines 171-174, 238-239, 464-473, and 752-753).

**12. Response to comment**: *3-2. As stated above, I am therefore very doubtful regarding all the patterns reported. If, as the authors appear to claim, that those patterns could be of ecological generality and help resolve different complexity-stability relationships in the literature, then I would expect to see the same patterns emerge from some alternative models where a basal resource is incorporated as a state variable, no matter as a species or a chemostat flux. It would be very helpful if the authors could proof this by conducting additional simulations with new models.*

**Response**: Thank you for your suggestions.

We have conducted sensitivity analyses on model structure (Appendix C in the supplementary material), with detailed descriptions incorporated into the main text. These modifications are reflected in Lines 752-753.

**13. Response to comment**: *3-3. Additionally, in the current model, interspecific and intraspecific competition are decoupled, too. For the exploiters, their intraspecific competition is modelled via a mutualist-dependent carrying capacity in the logistic term (though independently for each exploiter, determined by a shared parameter q), and interspecific competition as separated terms. However, these two should be coupled and modulated by the abundance of the basal resource, too. The current model without a basal resource therefore ignores the feedback created by a change in exploiters’ abundances to their own growth (via decreasing the resource). Such decoupling of resource-driven intraspecific and interspecific competition could create unrealistic coexistence purely due to stronger former than the latter and determined by the authors’ parameter chosen, while these two should coupled in scales via their mechanistic resource dependence. I thus have no much faith in the coexistence patterns revealed.*

**Response**: Thank you for your valuable comments.

Our previous work demonstrated that interspecific interference competition among fig wasps can reduce the virulence of fig wasps and stabilize the fig-wasp mutualism (Wang et al. 2009).

In the current study, we fixed the intraspecific competition intensity (α_0_ = 0.13) and analyzed changes in community stability under three distinct interspecific competition scenarios: low value (β = 0.1), intermediate value (β = 0.14), and high value (β = 0.16). These modifications are reflected in Lines 335-336, 344-345, and 349-350.

References:

Wang RW, Ridley J, Sun BF, Zheng Q, Dunn DW, et al. Interference Competition and High Temperatures Reduce the Virulence of Fig Wasps and Stabilize a Fig-Wasp Mutualism. PLOS ONE, 2009, 4(11): e7802.

**14. Response to comment**: *3-4. A relatively minor point: please clarify the direction of effect in parameter beta_ij, is it the effect of j on i, or the reverse? Since the whole simulation and analysis structure is mirroring the work by M. Kondoh, though with Kondoh’s annotation the direction of the effect seems to be the opposite of the current study. I am therefore concern if the interaction matrix is corrected coded (could not check the codes though, apologies---am not a matlab user).*

**Response**: Thank you for your suggestions.

In table 1, we set *β_ji_*	denotes interspeciﬁc competition of *F_j_* to *F_i_*, and *β_ji_* = β ∈ [0,0.18]. So, our model results of the full text are not affected by the direction of the interspecific competition (i.e., *β_ji_* or *β_ij_*).

**Thank you very much for your valuable comments and suggestions.**

**Reviewer #3: Comments**

**1. Response to comment**: *The present manuscript analyzes a model of a mutualist, exploiters and two types of predators (motivated by an empirical wasp system) using numerical simulations. In addition to the food web structure, the top predator adaptively forages on all other species. The manuscript first determines the stability diagram for the four species food web structure and then adds many exploiter types to the model that vary in growth rates and network connections. In this context, it is shown that the stability of the entire system can have several dependencies on the connectance of the food web, including non-monotonic relationships. Two related observations are presented. First, there are chaotic dynamics in the model, and second, a number of new feedback loops emerge when the top predator adaptively forages. Last, the authors argue that empirical data from freshwater and marine systems display some of the behavior they found through simulations.*

*I found the stability-connectance results counterintuitive and interesting. Similarly, the chaotic dynamics have a beautiful mathematical structure. In my view, the connection between these two sets of results needs to be made stronger. Because there are many processes in the model, it is difficult to know what is causing the new stability-connectance results. It would be useful to have some clearer “control” simulations in which we can build the counterintuitive results from each of the modeling ingredients. In the Technical Comments section below, I list a number of more specific comments related to this concern. The results in the manuscript could be connected to one another more clearly and the analysis of the empirical data is lacking. In addition, the readability of the manuscript could be improved. It can be difficult to follow exactly which parameters are being varied on a first reading. Similarly, it is difficult for the reader to build an intuition about what elements of the model are producing the results. The Readability Comments section has more specific comments describing these issues.*

**Response**: Thank you very much for your valuable comments. We have addressed each of your valuable comments point by point.

**2. Response to comment**: *TECHNICAL COMMENTS*

*As I understand it, the paper is arguing that adaptive foraging is causing new stability-connectance relationships via non-equilibrium dynamics, but the connections between these different sets of results is tenuous. First, it would be useful to have a panel in Fig. 3 without adaptive foraging to convince the reader that adaptive foraging is a crucial ingredient for the new behaviors. Is it possible to derive the behavior of a model without adaptive foraging analytically using the standard complexity-stability approach? Results for a simple model without adaptive foraging will provide a baseline for the reader to understand which behaviors come from which processes.*

**Response**: Thank you for your valuable comments.

We have analyzed stability changes with different adaptation strengths: Fig. 2 illustrates changes in system stability (i.e., local stability) under low (g=0.08), intermediate (g=0.28), and high (g=0.48) adaptation strengths for the four-species system, while Fig. 4 similarly analyzes stability variations (i.e., community persistence or robustness) in the general system under these three adaptation strengths.

In addition, in Fig. 5A and Fig 5B, varying the adaptation strength (g=0.08, 0.28, 0.48) only lightly change the curve shape of the connectance-stability relationship.

Therefore, we infer the complexity-stability relationship may be similar in the cases of g=0 and g=0.08.

**3. Response to comment**: *Second, if the authors intend to argue that the non-equilibrium behaviors they observe cause the surprising stability-connectance behaviors, there needs to be a stronger quantitative connection between them. For example, do the peaks in the non-monotonic behavior in Fig. 3 correlate with the frequency of simulations that are not at equilibrium or exhibit chaos? It might be instructive to analyze some simpler scenarios. For example, if every exploiter has the same growth rate and a simple network structure is chosen, perhaps it is possible to draw some analytical conclusions.*

**Response**: Thank you for your valuable suggestions.

Our theoretical results suggested that only the intermediate intensity of foraging adaptation can lead to chaos (case III; Fig. 2), while earlier theoretical and empirical work showed that chaos may provide a means for flexibility (Károlyi et al. 2000; Kosuta et al. 2008). In our system, an intermediate adaptation intensity of the top predator can produce the flexibility, which may facilitate the adaptation of the food web to environmental disturbance. Relevant descriptions are presented in Lines 436-442.

Whether the non-equilibrium behaviors cause the surprising stability-connectance modes need to analyze in future.

**References**

Károlyi G, Péntek Á, Scheuring I, Tél T, Toroczkai Z. Chaotic flow: the physics of species coexistence. Proceedings of the National Academy of Sciences. 2000;97(25):13661-5.

Kosuta S, Hazledine S, Sun J, Miwa H, Morris RJ, Downie JA, et al. Differential and chaotic calcium signatures in the symbiosis signaling pathway of legumes. Proceedings of the National Academy of Sciences. 2008;105(28):9823-8.

**4. Response to comment**: *From Equation (5) in the Methods, it appears that g cannot change the equilibrium solutions of the model, though it could affect stability. If this is true, then it is additional evidence that adaptive foraging creates non-equilibrium solutions.*

**Response**: Yes, the parameter *g* cannot change the equilibrium solutions of the model.

**5. Response to comment**: *Relatedly, it would be good to make a quantitative connection between the feedback loops proposed in Fig. 5 and the main results in Fig. 3. Is it possible to predict some of the qualitative behaviors from the feedbacks resulting from simulations? Fig. 2 also has potential implications for Fig. 3 that could be drawn out more. For example, higher interspecific competition tends to reduce coexistence (as expected) in both Fig. 2 and 3.*

**Response**: Thank you for your valuable comments.

In this study, we propose a potential qualitative mechanism underlying the complexity-stability relationships revealed in Fig. 4, that is, adaptive foraging by the top predator can modulate system stability by altering the dominant positive/negative feedback loops within the community (trait-mediated indirect effects), as elaborated in Lines 396-399, 425-427, and 515-518.

Furthermore, we delve deeper into the intrinsic linkages among adaptive foraging, positive-negative feedbacks, and multimodal complexity-stability relationships (see Lines 518-532).

Finally, we analyze the similar trends between model predictions and empirical data analyses in Fig. 5 and Appendix E in the supplementary material (see Lines 366-368, 373-375, and 379-381).

**6. Response to comment**: *The analysis of the empirical data is lacking. The manuscript provides no details about how or what models are fit. More importantly, there is not any model comparison. For example, does a quadratic (or whatever hump shaped curve is in Fig. 6D) fit better than a line in Fig 6C? Reciprocally, does a line fit better than a quadratic in Fig. 6D? A robust statistical approach is required here and it needs to be described clearly. Similarly, how are the connectances being modified in the data? Could they be changing with other important ecological parameters?*

**Response**: Thank you for your valuable comments.

Simulation analyses revealed that increasing interspecific competition intensity may shift the complexity-stability relationship from a monotonically decreasing pattern to a unimodal pattern (initially increasing then decreasing). These predictions align well with observed complexity-stability relationships in freshwater communities (characterized by low salinity and weak competition) versus marine communities (exhibiting high salinity and intense competition), as demonstrated in Fig. 5 (see Lines 357-361, 366-368, 373-375, 379-381, and Appendix E).

In addition, the stability and connectance of empirical data are derived from the recent work (Jacquet et al. 2016).

**References**

Jacquet C, Moritz C, Morissette L, Legagneux P, Massol F, Archambault P, et al. No complexity–stability relationship in empirical ecosystems. Nature Communications. 2016;7(1):1-8.

**7. Response to comment**: *The bifurcation diagrams in the Supplement are very beautiful. Are there other situations in which adaptive foraging produces these sorts of bifurcations?*

**Response**: Thank you very much.

Since the three adaptation strengths (g = 0.08, 0.28, 0.48) shown in Fig. 4 are already present in Fig. S1 of the earlier version, we have incorporated Supplementary Fig. S1 as Fig. 2 in the main text to maintain logical continuity. Therefore, chaotic dynamics of the four-species MEST community have been incorporated into Fig. 2, with corresponding descriptions in Lines 229-232 and 266-289.

For “other situations that adaptive foraging produces chaotic bifurcations”, we infer that it is true, because in Fig. S11 (Appendix D), it also shows the chaos of complex community.

**8. Response to comment**: *READABILITY COMMENTS*

*Including the Methods section before the Results would greatly improve the reader’s ability to keep track of what is being simulated. As it is currently written, the reader has to skip ahead to know even the model form.*

**Response**: Thank you for your valuable comments. We have placed the methods section earlier, which presented in Lines 159-262.

**9. Response to comment**: *In Equation (2), why does the strength of the benefit to the exploiter depend on the ratio of the mutualist to the exploiter? The biological explanation of this modeling choice is unclear. Presumably, it is describing the interaction between the mutualist, exploiter and resource, but I am not sure why the ratio is the correct term for this, rather than simply the mutualist abundance. Do other functional forms for this mutualism drastically change the results?*

**Response**: Thank you for your valuable comments.

First, we justify the exclusion of the basal resource (i.e., fig tree) in this adaptive network model through both qualitative arguments (i.e., the markedly longer life cycle of the fig tree compared to fig wasps and the predatory ant) and quantitative validation (structural sensitivity analyses; Appendix C), our adaptive network framework treats the mutualist as one potential resource (see Lines 171-174, 238-239, 464-473, and 752-753).

Then, we assume that carrying capacity as a function of density of interaction partners (May 1981; Addicott 1981; Wolin & Lawlor 1984).

**References**

Addicott, J.F. (1981). Stability properties of two-species models of mutualism: simu-lation studies. Oecologia, 49, 42–49.

May, R.M. (1981). Models for two interacting populations. In: Theoretical Ecology: Principles and Applications (ed. May, R.M.). Blackwell Scientiﬁc, Oxford, UK, pp. 49–70.

Wolin, C.L. & Lawlor, L.R. (1984). Models of facultative mutualism: density effects. Am. Nat., 124, 843–862.

**10. Response to comment**: *When varying the connectance in Fig. 3, exactly which links are being turned on and off? From the Methods section, it was unclear to me whether or not connectance was modifying any link in the food web, only those involving the top predator or only those involving the exploiters. Clearly, some of these functionally interactions are not equivalent to the others, complicating the definition of connectance.*

**Response**: Thank you for your valuable comments. We have added the descriptions presented in Lines 242-246.

**11. Response to comment**: *The Methods section would benefit from more discussion of the differential equations for theta. Is this functional form the same as other studies. It would be useful to point out that the equations constrain the sum of all theta values is constant throughout the dynamics.*

**Response**: Thank you for your valuable comments. In the figure legend of Fig. 1, we have set $\theta_{C}=1-\sum_{j=0}^{n} \theta_{j}$, which is presented in Line 180. In addition, in the four-species MEST community, the functional form of each preference trait can be read:

$\frac{d\theta_{0}}{dt}=g\theta_{0}\left[ e_{P}u_{0}F_{0}-e_{P}\left( \theta_{0}u_{0}F_{0}+\theta_{1}u_{1}F_{1}+\theta_{c}u_{c}C \right) \right]$,

$\frac{d\theta_{1}}{dt}=g\theta_{1}\left[ e_{P}u_{1}F_{1}-e_{P}\left( \theta_{0}u_{0}F_{0}+\theta_{1}u_{1}F_{1}+\theta_{c}u_{c}C \right) \right]$,

$\frac{d\theta_{C}}{dt}=-\frac{d\theta_{0}}{dt}-\frac{d\theta_{1}}{dt}$.

and the initial value must meet $\theta_{0}+\theta_{1}+\theta_{c}=1$ when t = 0 (Appendix C).

**12. Response to comment**: *Using the same color bar across panels in Fig. 2 would improve its clarity so a reader can see right away that some cases are always unstable.*

**Response**: Thank you for your valuable comments.

Chaotic dynamics of the four-species MEST community have been incorporated into Fig. 2, with corresponding descriptions in Lines 229-232 and 266-289; second, the color labels in Fig. 3 have been standardized and minor layout adjustments have been made to Fig. 4.

**Thank you very much for your valuable comments and suggestions.**

**Other changes:**

1. Line 2: we changed “a multiplex community with multiple interaction types” to “mutualist-exploiter-predator communities”.
2. Line 4: we added “Jia Li^2^”
3. Lines 13-14: we changed “^4^Hunan Institute of Agricultural Information and Engineering, Hunan Academy of Agricultural Sciences, Changsha 410125, China” to “^4^Hunan Institute of Agricultural Economics and Information, Hunan Academy of Agricultural Sciences, Changsha, 410125, Hunan, China.”.
4. Lines 34-57: we re-organized the abstract “Early ecological theory predicts that complex ecological networks are unstable and are unlikely to persist, despite many empirical studies of such complexity in nature. This inconsistency has fascinated ecologists for decades. To resolve the complexity-stability debate, coupling population dynamics and trait dynamics is considered to be an important way to understand the long-term stability of ecological community assemblages. However, we still do not know how eco-evolutionary feedbacks affect the relationship between complexity and stability in ecologically realistic networks with both antagonistic and mutualistic interactions. Here, we explored an adaptive network model to evaluate how the evolution of foraging preference to determine the relationship between network complexity (i.e., connectance) and stability (i.e., community persistence at steady state) in mutualist-exploiter-predator communities (MEST). Our theoretical results showed: (i) adaptive foraging of the top predator contributes to the stability of mutualism and intermediate intensity of foraging adaptations can lead to chaotic dynamics in a four-species MEST community; (ii) the complexity-stability relationship may show positive monotonic, negative monotonic, peaked and double-peaked patterns in general MEST communities, while the double-peaked pattern is only obtained when both the adaptation intensity and interspecific competition are high. Furthermore, model predictions may be consistent with both the negative monotonic pattern revealed in freshwater communities and the peaked pattern revealed in marine communities. Finally, we infer that foraging adaptations of the top predator may alter positive or/and negative feedback loops (trait-mediated indirect effects) to affect the stability of general MEST communities. Our adaptive network framework may provide an effective way to address the complexity-stability debate in real ecosystems.”
5. Lines 58-59: we changed “Adaptive foraging, mutualistic-antagonistic interactions, complexity-stability debate, chaos, positive-negative feedback, interspecific competition” to “Adaptive foraging, chaos, eco-evolutionary feedbacks, complexity-stability debate, mutualistic-antagonistic interactions, interspecific competition”.
6. Lines 66-67: we changed “predator-prey dynamics and” to “mutualist-exploiter-predator dynamics and”
7. Lines 70-71: we changed “identified various patterns in the complexity-stability relationship, indicating that both the intensity of foraging adaptation and competition among species play critical roles.” to “identified various complexity-stability relationship patterns, indicating that both the intensity of foraging adaptations and interspecific competition play critical roles”
8. Line 73: we changed “ecological communities and contribute to a more nuanced understanding of how ecosystems function and persist. Ultimately, our research offers” to “ecological communities. Ultimately, our adaptive network framework offers”
9. Line 105: we changed “In addition to different stability metrics (i.e., variability, resistance, resilience and persistence (4, 12, 17)) that could explain the inconsistent complexity-stability relationships described above, one potential reason for these inconsistencies could be” to “One potential reason for these variable complexity-stability relationships could be”
10. Lines 110-114: we changed “Incorporating trait dynamics into a single ecological framework can not only greatly increase the system dimension, but may change community stability (21-25). Foraging preference (26, 27), an important behaviour trait, and is thought to potentially help resolving the complexity-stability debate (28).” to “In fact, incorporating phenotypic trait dynamics into the single ecological framework can not only greatly increase the system dimension, but may change community stability (18-20). However, we still do not know how eco-evolutionary feedbacks affect the relationship between complexity and stability in ecologically realistic networks.”
11. Lines 115-116: we changed “Currently, ecologists have focused on the effects of foraging adaptation on community stability in food webs (29-32) or mutualistic networks (33-35).” to “Adaptive foraging (21), an important behaviour trait, and is thought to potentially affect the stability of food webs (22, 23) and mutualistic networks (24, 25).”
12. Line 125: we changed “with no” to “without the”
13. Lines 130-132: we changed “However, little is known about the community dynamics due to the AF in a hybrid network with multiple interaction types.” to “However, little is known about community dynamics driven by the AF in real ecosystems with both mutualistic and antagonistic interactions.”
14. Line 133: we changed “a framework for simple omnivorous food webs that incorporate” to “theoretical framework that incorporates”
15. Lines 134-135: we changed “The framework includes four species and crosses three trophic levels:” to “The model framework includes four species across three trophic levels”
16. Line 138: we changed “In addition” to “Moreover”
17. Line 144: we changed “, we assumed” to “; meanwhile, it”
18. Lines 147-149: we changed “However, it is still unclear how the AF of the top predator affects the relationship between network structure and stability in natural communities.” to “However, it is still unclear how adaptive foraging of the top predator affects the complexity-stability relationship in mutualist-exploiter-specialist predator-top predator (MEST) communities.”
19. Lines 150-157: we changed “In what follows, we establish an adaptive network framework to evaluate how the evolution of foraging preference (behaviour trait) to determine the relationship between network complexity (i.e., connectance) and stability (i.e., community persistence at steady state) in a multiplex community (MEST: mutualist-exploiter-specialist predator-top predator) with multiple interaction types. This study focuses on answering two key scientific questions: (i) what are the intrinsic mechanisms (e.g., trait-mediated indirect effects) by which foraging adaptation alters community stability (community persistence) and (ii) how to resolve inconsistent relationships between network complexity (e.g., connectance) and stability in model and empirical ecosystems. Our theoretical work may provide an effective way to reconcile the complexity-stability debate in ecosystems.” to “In what follows, we establish an adaptive network model to evaluate the effects of the adaptive foraging on the relationship between network complexity (i.e., connectance) and stability (i.e., community persistence at steady state) in MEST communities. This study mainly focuses on answering two key scientific questions: (i) what are the intrinsic mechanisms by which variable complexity-stability relationship emerges in an adaptive dynamic framework and (ii) how to reconcile the complexity-stability debate in model and empirical ecosystems. Our work may offer valuable insights on the stability of complex ecological networks.”
20. Line 198: we added “and function WP defines the fitness (39) of P.”
21. Lines 206-207: we added “similar to early viewpoint that carrying capacity as a function of density of interaction partners (40, 41),”
22. Lines 216-217: we deleted “In Eq. (4), *W_P_*$=\frac{{dP}/{dt}}{P}$, defines the fitness (81) of *P*, and the fitness gradient is $\frac{\partial W_{P}}{\partial\theta_{j}}=e_{P}\left( u_{j}F_{j}-u_{C}C \right)$. Then, we can obtain $\frac{d\theta_{j}}{dt}=g{e_{P}\theta}_{j}\left( \left( 1-\theta_{j} \right)\left( u_{j}F_{j}-u_{C}C \right)-\sum_{k\in\Omega} \theta_{k}\left( u_{k}F_{k}-u_{C}C \right) \right)$, *j=0,1,2,…,n.* (6)

where $\Omega$ presents all prey resources of the top predator *P* other than *F_j_*.”.

23. Lines 553-555: we changed “We thank Ellen van Velzen and Peter Abrams for helpful comments on an earlier version of this manuscript. This work was supported by the National Natural Science Foundation of China (32201259, 32271548, 32201264), NSFC-Yunnan United fund (U2102221) and Jiangxi Provincial Natural Science Foundation (20224BAB215010).” to “We express our sincere gratitude to editors and three anonymous reviewers for their valuable comments and insights on this manuscript; we also thank Ellen van Velzen and Peter Abrams for helpful comments on an earlier version of this manuscript.”
24. Lines 570-577: we reorganized “L.W.: Conceptualization, Funding Acquisition, Methodology, Writing – Original Draft Preparation, Writing – Review & Editing; T.W.: Methodology, Software, Visualization, Writing – Review & Editing; X.-W.Z.: Data Curation, Visualization, Writing – Review & Editing; X.-F.L.: Methodology, Software, Visualization, Writing – Review & Editing; J.L.: Methodology, Software, Visualization, Writing – Review & Editing; J.-B.L.: Conceptualization, Funding Acquisition, Supervision, Writing – Review & Editing; W.-R.W.: Conceptualization, Funding Acquisition, Supervision, Writing – Review & Editing.”
