# Supplementary material for: Evolution of foraging behaviour induces variable complexity-stability relationships in mutualist-exploiter-predator communities
Source: PLoS Comput Biol. 2025 Jul 9;21(7):e1013245. doi: 10.1371/journal.pcbi.1013245 (PMC12240360; doi:10.1371/journal.pcbi.1013245)
Supplement: Attachment — Submitted filename: Response_to_Reviewers_auresp_2.docx [file pcbi.1013245.s009.docx]

**List of Responses**

Dear Editor:

Thank you again for your comments related to our manuscript entitled “Evolution of foraging behaviour induces variable complexity-stability relationships in mutualist-exploiter-predator communities” (Ms. Ref. No.: PCOMPBIOL-D-24-01756R1). Those comments are all valuable and very helpful for revising and improving our paper, as well as an important guiding significance to our researches. We have studied the comments carefully and have edited the manuscript to meet with the comments. Revised parts are marked in red in the paper.

The main corrections in the paper and the response to the comments of editors are as following:

**Response to the editors’ comments:**

**1. Response to comment**: ** Appendix B varies one parameter at a time. Please add a multivariate exploration (e.g. Latin-hyper-cube sampling with partial-rank correlations) across all free parameters to demonstrate that the main complexity–stability patterns are robust to joint uncertainty. A concise summary figure or table would be sufficient.*

**Response**: Thank you for your valuable comment. We have employed the Latin Hypercube Sampling-Partial Rank Correlation Coefficient (LHS-PRCC) method for sensitivity analyses (Marino et al., 2008), and reorganized the results of the parameter sensitivity analysis in Appendix B, which are listed as follows:

By setting t = 2000, we can obtain the significant influence of key parameters on the state variables (Fig. S5). In Fig. S5A, parameters $r_{0}$, $\beta$, $u_{C}$, $\alpha_{0}$, $u_{0}$, $a_{C}$ and $r_{1}$ exhibit pronounced influences on F0 (*p* < 0.01; table S1). Specifically, parameters $r_{0}$, $\beta$ and $u_{C}$ promote the increase of F0, with their effect magnitudes ranked as $\left| PRCC\left( r_{0} \right) \right|>\left| PRCC\left( \beta\right) \right|>\left| PRCC\left( u_{C} \right) \right|$; while the parameters $\alpha_{0}$, $u_{0}$, $a_{C}$ and $r_{1}$ inhibit the growth of F0, following the hierarchy $\left| PRCC\left( r_{1} \right) \right|>\left| PRCC\left( a_{C} \right) \right|>\left| PRCC\left( u_{0} \right) \right|>\left| PRCC\left( \alpha_{0} \right) \right|$. In Fig. S5B, the parameters $u_{0}$, $a_{C}$ and $r_{1}$ promote the increase of F1, with the order of effect magnitude being $\left| PRCC\left( r_{1} \right) \right|>\left| PRCC\left( u_{0} \right) \right|>\left| PRCC\left( a_{C} \right) \right|$; while the parameters $u_{1}$ and β inhibit the increase of F1, where $\left| PRCC\left( \beta\right) \right|>\left| PRCC\left( u_{1} \right) \right|$. In Fig. S5C, the parameters $r_{0}$, β, and $u_{1}$ promote the increase of C, with the magnitude of effects following $\left| PRCC\left( r_{0} \right) \right|>\left| PRCC\left( \beta\right) \right|>\left| PRCC\left( u_{1} \right) \right|$; while the parameters $\alpha_{0}$ and $r_{1}$ inhibit the increase of C, where $\left| PRCC\left( r_{1} \right) \right|>\left| PRCC\left( \alpha_{0} \right) \right|$. In Fig. S5D, the parameters $r_{0}$, $\beta$, $u_{C}$, $e_{P}$, and $u_{1}$ promote the increase of P, with the magnitude of effects following $\left| PRCC\left( r_{0} \right) \right|>\left| PRCC\left( \beta\right) \right|>\left| PRCC\left( u_{C} \right) \right|>\left| PRCC\left( e_{P} \right) \right|>\left| PRCC\left( u_{1} \right) \right|$; while the parameters $d_{P}$, $a_{C}$, $\alpha_{P}$, $r_{1}$, and $\alpha_{0}$ inhibit the increase of P, where $\left| PRCC\left( \alpha_{0} \right) \right|>\left| PRCC\left( r_{1} \right) \right|>\left| PRCC\left( \alpha_{P} \right) \right|>\left| PRCC\left( a_{C} \right) \right|>\left| PRCC\left( d_{P} \right) \right|$. In Fig. S5E, the parameters $u_{0}$, $r_{0}$, $e_{P}$, $\alpha_{C}$, $\beta$, and $u_{1}$ promote the increase of $\theta_{0}$, with the magnitude of effects following$\left| PRCC\left( u_{0} \right) \right|>\left| PRCC\left( r_{0} \right) \right|>\left| PRCC\left( e_{P} \right) \right|>\left| PRCC\left( \alpha_{C} \right) \right|>\left| PRCC\left( \beta\right) \right|>\left| PRCC\left( u_{1} \right) \right|$; while the parameters $d_{P}$, $\alpha_{0}$, $\alpha_{P}$, $e_{C}$, $r_{1}$, and $a_{C}$ inhibit the increase of $\theta_{0}$, where $\left| PRCC\left( a_{C} \right) \right|>\left| PRCC\left( r_{1} \right) \right|>\left| PRCC\left( e_{C} \right) \right|>\left| PRCC\left( \alpha_{P} \right) \right|>\left| PRCC\left( \alpha_{0} \right) \right|>\left| PRCC\left( d_{P} \right) \right|$. In Fig. S5F, the parameters $u_{1}$, $\alpha_{0}$, $r_{1}$, and $a_{C}$ promote the increase of $\theta_{1}$, with the magnitude of effects following $\left| PRCC\left( r_{1} \right) \right|>\left| PRCC\left( \alpha_{0} \right) \right|>\left| PRCC\left( a_{C} \right) \right|>\left| PRCC\left( u_{1} \right) \right|$; while the parameters $u_{C}$, $r_{0}$, and $\beta$ inhibit the increase of $\theta_{1}$, where $\left| PRCC\left( r_{0} \right) \right|>\left| PRCC\left( \beta\right) \right|>\left| PRCC\left( u_{c} \right) \right|$.

Fig. S5 Sensitivity analysis using PRCC at t=2000. (A) PRCC-F0; (B) PRCC-F1; (C) PRCC-C; (D) PRCC-P; (E) PRCC-θ_0_; (F) PRCC-θ_1_.

**Reference**

Marino, S., Hogue, I.B., Ray, C.J., Kirschner, D.E. A methodology for performing global uncertainty and sensitivity analysis in systems biology. Journal of Theoretical Biology. 2008;254(1):178-196.

**2. Response to comment**: ** The qualitative match between model predictions and published food-web patterns in Fig 5 is still weak validation at best - please revise the discussion to acknowledge this more directly with suitable caveats, or provide a stronger validation (may not be possible).*

**Response**: Thank you very much for your comment. We have adopted your suggestion and redescribed "Our theoretical predictions may match empirical results to a certain extent" presented in Line 492.

**3. Response to comment**: ** The manuscript still exceeds 9,000 words and contains several minor grammatical slips (e.g. “biomss”, “responds” instead of “responses”).*

**Response**: Thank you for your comments. We have changed “biomss” to “biomass” presented in Line 273, and also changed “responds” to “responses”. Moreover, we re-counted the word count of the main text (i.e., Manuscript document), totaling 7,983 words, including 1,699 words of the references.

**4. Response to comment**: *Figure resubmission: While revising your submission, please upload your figure files to the Preflight Analysis and Conversion Engine (PACE) digital diagnostic tool, https://pacev2.apexcovantage.com/. PACE helps ensure that figures meet PLOS requirements. To use PACE, you must first register as a user. Registration is free. Then, login and navigate to the UPLOAD tab, where you will find detailed instructions on how to use the tool. If you encounter any issues or have any questions when using PACE, please email PLOS at figures@plos.org. Please note that Supporting Information files do not need this step. If there are other versions of figure files still present in your submission file inventory at resubmission, please replace them with the PACE-processed versions.*

**Response**: Thank you for your valuable comments. We made pixel corrections to all six figures in the main text based on your suggestions.

**5. Response to comment**: *Reproducibility: To enhance the reproducibility of your results, we recommend that authors of applicable studies deposit laboratory protocols in protocols.io, where a protocol can be assigned its own identifier (DOI) such that it can be cited independently in the future. Additionally, PLOS ONE offers an option to publish peer-reviewed clinical study protocols. Read more information on sharing protocols at https://plos.org/protocols?utm_medium=editorial-email&utm_source=authorletters&utm_campaign=protocols*

**Response**: Thank you for your suggestions. Our work falls within the scope of ecological theory research, and we have shared all the simulation codes (Appendix F).

**Thank you very much for your valuable comments and suggestions.**

**Other changes:**

1. Line 321: we changed “Figs. S4-S9” to “Figs. S4-S5”.
2. Line 432: we changed “Fig. S11” to “Fig. S7”.
3. Line 467: we changed “Fig. S10” to “Fig. S6”.
4. Lines 557-562: we changed “L.W. acknowledges the support of both National Natural Science Foundation of China (NSFC; 32201259) and Jiangxi Provincial Natural Science Foundation (20224BAB215010); X.-W.Z. acknowledges the support of the NSFC (32201264); J.-B.L. acknowledges the support of the NSFC (32271548) and W.-R.W. acknowledges the support of NSFC-Yunnan United fund (U2102221).” to “L.W. acknowledges the support of both National Natural Science Foundation of China (32201259) and Natural Science Foundation of Jiangxi Province (20224BAB215010); X.-W.Z. acknowledges the support of the National Natural Science Foundation of China (32201264); J.-B.L. acknowledges the support of the National Natural Science Foundation of China (32271548) and W.-R.W. acknowledges the support of National Natural Science Foundation of China-Yunnan Joint Fund (U2102221)”.
